# Kdm6a-CNN1 axis orchestrates epigenetic control of trauma-induced spinal cord microvascular endothelial cell senescence to balance neuroinflammation for improved neurological repair

**DOI:** 10.1038/s41413-024-00323-x

**Published:** 2024-03-25

**Authors:** Chengjun Li, Tian Qin, Jinyun Zhao, Yuxin Jin, Yiming Qin, Rundong He, Tianding Wu, Chunyue Duan, Liyuan Jiang, Feifei Yuan, Hongbin Lu, Yong Cao, Jianzhong Hu

**Affiliations:** 1 Department of Sports Medicine, Xiangya Hospital, Central South University, Xiangya Road 87, Changsha, 410008 China; 2 Key Laboratory of Organ Injury, Aging and Regenerative Medicine of Hunan Province, Xiangya Road 87, Changsha, 410008 China; 3 National Clinical Research Center for Geriatric Disorders, Xiangya Hospital, Central South University, Xiangya Road 87, Changsha, 410008 China; 4 Department of Spine Surgery and Orthopaedics, Xiangya Hospital, Central South University, Xiangya Road 87, Changsha, 410008 China

**Keywords:** Neurophysiology, Pathogenesis

## Abstract

Cellular senescence assumes pivotal roles in various diseases through the secretion of proinflammatory factors. Despite extensive investigations into vascular senescence associated with aging and degenerative diseases, the molecular mechanisms governing microvascular endothelial cell senescence induced by traumatic stress, particularly its involvement in senescence-induced inflammation, remain insufficiently elucidated. In this study, we present a comprehensive demonstration and characterization of microvascular endothelial cell senescence induced by spinal cord injury (SCI). Lysine demethylase 6A (Kdm6a), commonly known as UTX, emerges as a crucial regulator of cell senescence in injured spinal cord microvascular endothelial cells (SCMECs). Upregulation of UTX induces senescence in SCMECs, leading to an amplified release of proinflammatory factors, specifically the senescence-associated secretory phenotype (SASP) components, thereby modulating the inflammatory microenvironment. Conversely, the deletion of UTX in endothelial cells shields SCMECs against senescence, mitigates the release of proinflammatory SASP factors, and promotes neurological functional recovery after SCI. UTX forms an epigenetic regulatory axis by binding to calponin 1 (CNN1), orchestrating trauma-induced SCMECs senescence and SASP secretion, thereby influencing neuroinflammation and neurological functional repair. Furthermore, local delivery of a senolytic drug reduces senescent SCMECs and suppresses proinflammatory SASP secretion, reinstating a local regenerative microenvironment and enhancing functional repair after SCI. In conclusion, targeting the UTX-CNN1 epigenetic axis to prevent trauma-induced SCMECs senescence holds the potential to inhibit SASP secretion, alleviate neuroinflammation, and provide a novel treatment strategy for SCI repair.

## Introduction

Spinal cord injury (SCI) is a severe traumatic disorder of the central nervous system (CNS), typically leading to irreversible loss of both locomotor and sensory functions. The repercussions of SCI extend beyond the physical realm, severely impacting the quality of life for affected individuals, often resulting in disabilities that pose risks to their overall well-being. The economic burden associated with SCI is substantial, placing a considerable financial strain on both affected families and the broader social healthcare system.^1,2^ Following SCI, the injured cells experience morphological, physiological, chemical, and immune system alterations that impede regeneration. Clinical strategies for SCI intervention primarily encompass early surgical decompression, administration of high-dose glucocorticoids, anti-inflammatory therapy, and neural rehabilitation training. Despite these efforts, there remains a notable absence of effective treatment modalities to facilitate the recovery and regeneration of the injured spinal cord.^2^

Despite significant progress in unraveling the molecular intricacies following spinal cord injury (SCI) in mammals, therapeutic breakthroughs remain elusive. This discrepancy indicates that the comprehensive understanding of the pathological alterations occurring post-SCI is still incomplete. Following SCI, the immediate rupture of the blood-spinal cord barrier (BSCB) facilitates the extravasation of immune cells, contributing to the establishment of a proinflammatory milieu. SCI is further characterized as an inflammatory state driven by infiltrating macrophages and activated astrocytes.^3^ Inflammation represents one of the evolutionarily conserved mechanistic foundations of aging. Numerous age-related diseases manifest the accumulation of senescent cells. Despite their non-proliferative nature, senescent cells possess the capability to influence the microenvironment, establishing a close correlation with inflammatory status through the production of inflammatory mediators.^4^ Yet, in traumatic central nervous system (CNS) diseases, cellular senescence is frequently disregarded. Endothelial cells play a vital role in functional interactions with other cells, including pericytes, astrocytes, oligodendrocytes, neurons, etc., contributing to the formation of the neurovascular unit (NVU) and the maintenance of normal CNS function.^5,6^ Endothelial cells construct the vascular networks that supply the CNS, featuring a specialized structure crucial for preserving homeostasis and facilitating neurological functional repair.^7^ It has been reported that vascular endothelial cells underwent senescence in the degenerative and aging disease. The disruption of tight junctions in senescent endothelial cells results in increased vascular permeability and impairment of normal vascular function.^8,9^ However, whether vascular endothelial cells undergo cellular senescence in the trauma-induced pathophysiological process of spinal cord injury (SCI) and whether senescent cells secrete proinflammatory senescence-associated secretory phenotype (SASP) factors that impact neurological functional repair is worthy of further exploration.

Senescent cells usually express several senescence biomarkers including P53, P21, P16, senescence related β-galactosidase (SA-β-Gal). An important characteristic of senescent cells is the SASP, which involves the release of a diverse array of extracellular modulators, including cytokines, chemokines, growth factors, and proteases.^10,11^ Through the release of SASP factors, senescence could modulate local microenvironment and affect the neighboring cells. Notably, senescent cells that are induced by different stress stimuli may exhibit distinct SASP components.^12,13^ Accumulating evidence indicates that epigenetic dysregulation plays a crucial role as an essential driver for cellular senescence.^14^ Epigenetic modifications, encompassing chromatin remodeling, DNA methylation, and histone alterations, have the capacity to activate or repress cellular senescence in response to a variety of stimuli.^15,16^ The diverse nature of epigenetic changes in senescence precludes the identification of a universal phenotype for senescent cells. Histone deacetylase 1/2 (Hdac1/2) has been reported to maintain normal glomerular filtration function. Specific Hdac1/2 deletion could induce podocyte senescence, which generated a high amount of SASP including pro-inflammatory cytokines to damage podocyte, ultimately resulting in increased proteinuria and progressive renal failure.^17^ Ezh2, a histone methylase, has been found to stimulate Foxo1 expression by targeting H3K27me3. This process results in a decrease in the antioxidant potential of bone marrow stromal cells (BMSCs) and contributes to the senescence of BMSCs.^18^ Lysine demethylase 6A(KDM6A), also known as ubiquitously transcribed tetratricopeptide repeat, X chromosome (UTX), has been demonstrated to regulate cell senescence in non-traumatic diseases by specifically removing the dimethyl and trimethyl marks on H3K27.^19,20^ UTX plays a crucial role in regulating senescence-related genes in the hematopoietic system through both demethylase-dependent and non-dependent mechanisms.^21^ It is highly recommended to explore the typical epigenetic factors that regulate trauma-induced endothelial cell senescence and the mechanisms by which senescent cells induce neuroinflammatory features in the injured spinal cord.

Accumulation of persistent senescence in the injured spinal cord has been demonstrated to be detrimental to functional repair.^22^ Senolytic treatments have demonstrated efficacy in pre-clinical mouse experiments by eliminating senescent cells, thereby delaying, preventing, or alleviating various age- and senescence-related diseases.^23^ Senolytic drugs that have been tested include dasatinib (D, an FDA-approved tyrosine kinase inhibitor), quercetin (Q, a flavonoid present in many fruits and vegetables), A1155463 (Bcl-2 pro-survival family inhibitors), and fisetin (F, a flavonoid).^24^ Dasatinib and quercetin (D + Q) represent first-generation senolytics that have been investigated in preclinical models of aging and various diseases, encompassing bone, muscle, and neurological conditions.^25^ In obese mice, the combination of dasatinib and quercetin (D + Q) has been observed to decrease neuroinflammation, enhance neurogenesis markers, and reduce anxiety.^26^ Given the accumulating presence of senescent cells in traumatic SCI, D + Q may exert a positive therapeutic effect. However, to date, the impact of D + Q treatment on promoting neurological functional repair after SCI has been scarcely investigated. Since SCI-induced senescent cells are situated deep within the neuroparenchyma and systemic, intermittent administration of molecular compounds may not readily cross the BSCB, local administration of senolytics appears to be a more promising approach.

In the current study, we first characterized the phenotype of trauma-induced senescence in SCMECs and thoroughly explored changes in epigenetic factors in senescent SCMECs after SCI. Our findings revealed a significant upregulation of the epigenetic factor UTX in SCMECs after SCI, and its ability to target calponin 1 (CNN1) to form an epigenetic regulatory axis. Deletion of the UTX/CNN1 axis resulted in a reduction of trauma-induced senescence in SCMECs, restoration of vascular function to balance neurological inflammation, and improvement in neurological functional repair. Additionally, we observed that the local administration of senolytics (D + Q) treatment could effectively reduce the accumulation of senescent SCMECs, attenuate SASP secretion, and create a pro-regenerative microenvironment for SCI repair.

## Results

### Trauma induced vascular endothelial cell senescence in injured spinal cord

**Fig. 1:**
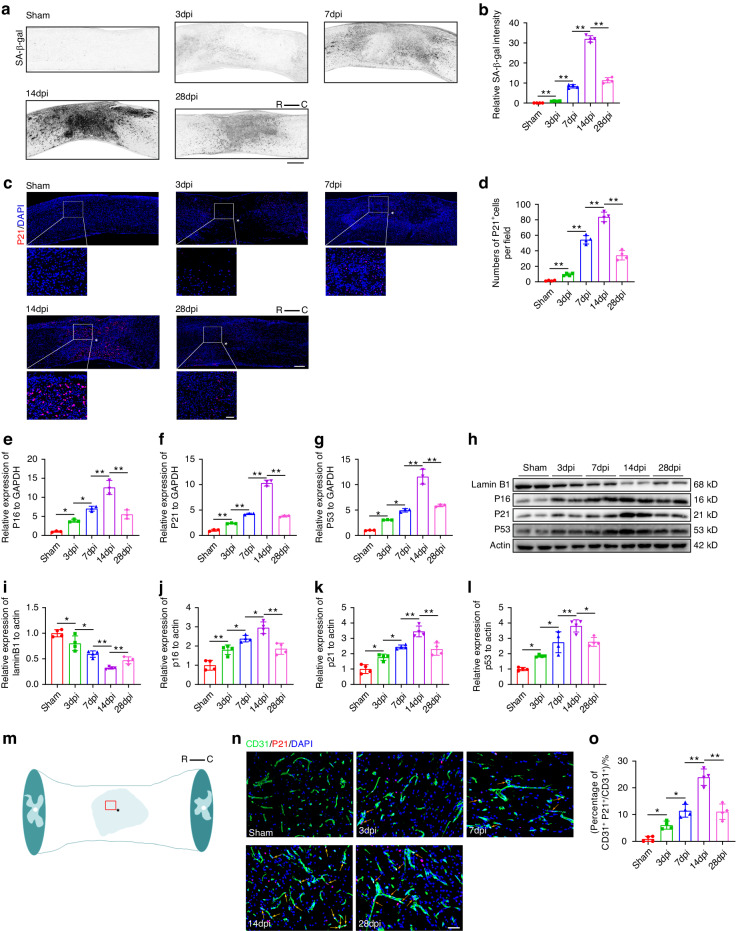
Trauma induced microvascular endothelial cell senescence in injured spinal cord. **a** SA-β-gal staining of spinal cord before injury and at 3 days, 7 days, 14 days, 28 days post SCI (dpi). Scale bar: 500 μm. R represents rostral, C represents caudal. **b** Quantification of relative SA-β-gal intensity in (**a**) (*n* = 4). **c** Representative images of P21 staining at different timepoints after SCI. Scale bar: 200 μm. Scale bar for magnified images: 50 μm. The asterisk indicates the lesion center. R represents rostral, C represents caudal. **d** Quantification of the number of P21^+^ cells in (**b**) (*n* = 4). **e** Relative expression of P16 expression at different timepoints after SCI by qRT-PCR (*n* = 3). **f** Relative expression of P21 expression at different timepoints after SCI by qRT-PCR (*n* = 3). **g** Relative expression of P53 expression at different timepoints after SCI by qRT-PCR (*n* = 3). **h** Western blot analysis of Lamin B1, P16, P21, and P53 expression at different timepoints after SCI. **i** Quantification of relative expression of Lamin B1 in (**h**) (*n* = 4). **j** Quantification of relative expression of P16 in (**h**) (*n* = 4). **k** Quantification of relative expression of P21 in (**h**) (*n* = 4). **l** Quantification of relative expression of P53 in (**h**) (*n* = 4). **m** A schematic diagram depicting the selective region of interest in (**n**). The asterisk denotes the injury center, while the red box indicates the region of interest. “R” represents rostral, and “C” represents caudal. **n** Representative images of CD31 (green) and P21 (red) staining at different timepoints after SCI. Scale bar: 50 μm. The orange arrow indicates CD31^+^P21^+^ cell. **o** Quantification of percentages of CD31^+^P21^+^/CD31^+^ cells in (**n**) (*n* = 4). Data are presented as the mean ± SD. *P < 0.05, **P < 0.01

To explore the potential induction of cell senescence by spinal cord injury (SCI), we established a contusion SCI model and utilized two well-recognized senescence markers, SA-β-gal and P21, to assess senescence levels at different time points. The SA-β-gal intensity showed an increase after SCI, reaching its peak 14 days post-injury and subsequently decreasing (Fig. 1a, b). Immunofluorescence staining demonstrated a significant rise in P21-positive cells in the injured spinal cord, peaking at 14 days post-SCI and then declining (Fig. 1c, d). We further employed quantitative real-time polymerase chain reaction (qRT-PCR) and Western blotting (WB) to examine the expression changes of senescent biomarkers in the spinal cord at different post-injury time points. Both the transcriptional and protein levels of P53, P21, and P16 increased initially, peaked on the 14th day after injury, and subsequently declined. Additionally, we observed that the expression pattern of Lamin B1 was opposite and significantly down-regulated after SCI, reaching its lowest level on day 14 after injury (Fig. 1e–l). We further investigated the senescence of different cell types after SCI through immunofluorescence staining. As depicted in Fig. S1, senescent cells were scarce in the area far from the center of the injury. However, in the central area of the injury, senescent cells were present, predominantly consisting of vascular endothelial cells and macrophages, accompanied by a small number of neural stem cells and astrocytes. Subsequently, we explored the phenotypes of spinal cord microvascular endothelial cells (SCMECs) senescence at different time points post-injury by co-localizing P21 with CD31 (a vascular endothelial cell marker) (Fig. 1m). SCMECs senescence occurred on the 3rd day post-SCI. The number of CD31^+^P21^+^ cells (senescent endothelial cells) increased continuously, reaching a peak on the 14th day after injury and then declining (Fig. 1n, o). These findings indicate that SCI induces vascular endothelial cell senescence in the injured spinal cord, manifesting distinctive senescence properties.

### Cellular senescence impaired endothelial cell functional activity

**Fig. 2:**
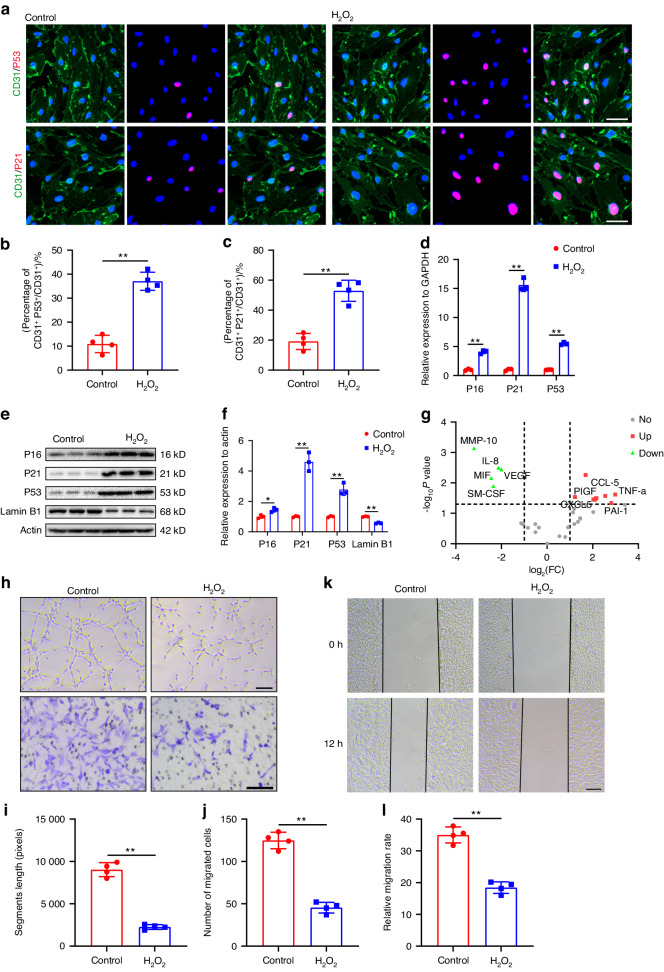
Cellular senescence impaired endothelial cell functional activity. **a** Representative images of CD31 (green) and P53 (red), CD31 (green) and P21 (red) in control and H_2_O_2_ treated bEnd.3 cells. Scale bar: 20 μm. **b** Quantification of percentages of CD31^+^P53^+^/CD31^+^ cells in (**a**) (*n* = 4). **c** Quantification of percentages of CD31^+^P21^+^/CD31^+^ cells in (**a**) (*n* = 4). **d** Relative expression of P16, P21 and P53 detected by qRT-PCR in control and H_2_O_2_ treated bEnd.3 cells (*n* = 3). **e** Western blot analysis of Lamin B1, P16, P21 and P53 expression in control and H_2_O_2_ treated bEnd.3 cells. **f** Quantification of relative expression of Lamin B1, P16, P21 and P53 in (**e**) (*n* = 3). **g** Volcano plot of PCR array for SASP factors in control and H_2_O_2_ treated bEnd.3 cells (*n* = 3). Red dots represent up-regulated genes. Green dots indicate down-regulated genes. **h** Representative images of bEnd.3 canalization (upper panel) and transwell migration (lower panel) in control and H_2_O_2_ treated bEnd.3 cells. Scale bar: 100 μm. **i** Quantification of segments lengths in (**h**) (*n* = 4). **j** Quantification of migrated cells in (**h**) (*n* = 4). **k** Representative images of horizontal migration in control and H_2_O_2_ treated bEnd.3 cells. Scale bar: 200 μm. **l** Quantification of relative migration rate in (**k**) (*n* = 4). Data are presented as the mean ± SD. *P < 0.05, **P < 0.01

Stress-induced cell senescence was induced in mouse brain microvascular endothelial cell line (bEnd.3 cells) through treatment with H_2_O_2_, and the senescent phenotype was characterized. Various concentrations of H_2_O_2_ resulted in elevated levels of SA-β-gal staining, accompanied by positive staining for P21 and P53 in endothelial cells (ECs) compared to the control group. Notably, the treatment with 200 μmol/L H_2_O_2_ exhibited the highest expression levels of senescent markers in ECs, establishing it as the optimal concentration for inducing cellular senescence (Fig. S2). The percentages of P53 and P21 positive cells increased in bEnd.3 cells treated with 200 μmol/L H_2_O_2_ (Fig. 2a–c). To validate these findings, primary brain endothelial cells (BMECs) were also treated with 200 μmol/L H_2_O_2_, resulting in increased percentages of P53 and P21 positive cells (Fig. S3). Additionally, in the cell line model, the expression levels of senescent markers were detected by qRT-PCR and WB. Compared with the control group, the expressions of P16, P21, and P53 genes were significantly elevated in bEnd.3 cells treated with 200 μmol/L H_2_O_2_ (Fig. 2d–f). PCR-array analysis revealed changes in the SASP expression profile in bEnd.3 cells treated with H_2_O_2_, showing increased expression of TNF-α, PAI-1, and CCL-5, while the expression of IL-8, VEGF, and MMP10 decreased (Fig. 2g). Given the observed senescent features in the cell model, we investigated how the accumulation of senescent vascular cells affected their own function in vitro. Compared to the control group, H_2_O_2_ administration led to a decrease in segment length and a significant reduction in the number of migrated bEnd.3 cells (Fig. 2h–j). The migration ability of bEnd.3 cells was also significantly reduced after H_2_O_2_ intervention, as shown in Fig. 2k, l. These results confirm the functional compromised in ECs associated with cell senescence.

### Epigenetic factor UTX exhibited upregulated characteristic features in ECs

**Fig. 3:**
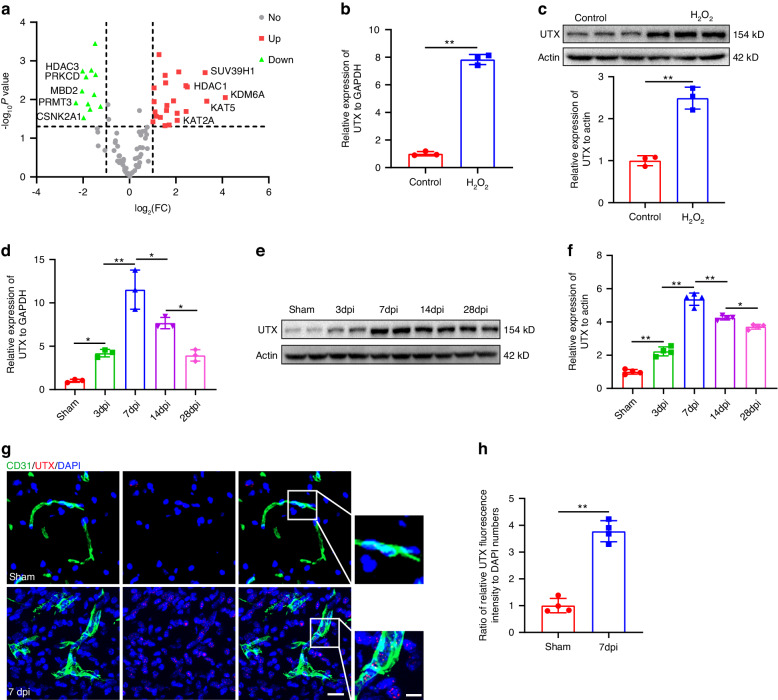
Epigenetic factor UTX exhibited upregulated characteristic features in ECs. **a** Volcano plot of PCR array for epigenetic factors in control and H_2_O_2_ treated BMECs (*n* = 3). Red dots represent up-regulated genes. Green dots indicate down-regulated genes. **b** Relative expression of UTX in control and H_2_O_2_ treated bEnd.3 cells detected by qRT-PCR (*n* = 3). **c** Western blot analysis and quantification of UTX expression in control and H_2_O_2_ treated bEnd.3 cells (*n* = 3). **d** Relative expression of UTX in spinal cord at different timepoints after SCI by qRT-PCR (*n* = 3). **e** Western blot of UTX expression in spinal cord at different timepoints after SCI. **f** Quantification of (**e**) (*n* = 4). **g** Representative fluorescent images of CD31 (green) and UTX (red) in sham and 7dpi groups. Scale bar: 20 μm. Scale bar for magnified images: 5 μm. **h** Quantification of (**g**) (*n* = 4). Data are presented as the mean ± SD. *P < 0.05, **P < 0.01

Previous studies have suggested that epigenetic factors might exert a regulatory role in the cellular senescence process.^27^ We isolated BMECs to establish a cell senescence model using H_2_O_2_. Subsequently, we conducted a PCR-array of the epigenetic enzyme profile to characterize changes in epigenetic factors during EC senescence. Under H_2_O_2_ treatment, BMECs underwent a stress response leading to cell senescence, accompanied by alterations in the epigenetic landscape. Notably, KDM6A, also known as UTX, exhibited the most significant increase in ECs (Fig. 3a). The mRNA and protein expression levels of UTX were also significantly elevated after H_2_O_2_ treatment in bEnd.3 cells, as depicted in Fig. 3b, c. To explore whether such changes in the transcriptional signatures of epigenetic factors also occurred in vivo, mice were subjected to injury to induce a SCI model. Interestingly, expression analysis of the entire injured spinal cord tissue revealed a marked upregulation in UTX, reaching a peak on the 7th day after injury, at both the mRNA and protein levels (Fig. 3d–f). Importantly, SCMECs responded to the injury by exhibiting a noticeable increase in UTX expression in the injured spinal cord tissue (Fig. 3g, h). Collectively, these findings suggest a potential mechanistic association between UTX and senescence in endothelial-specific cells, observed in both cellular and animal models.

### Conditional knockout of UTX in ECs impaired cell senescence and facilitated neurological recovery post SCI

**Fig. 4:**
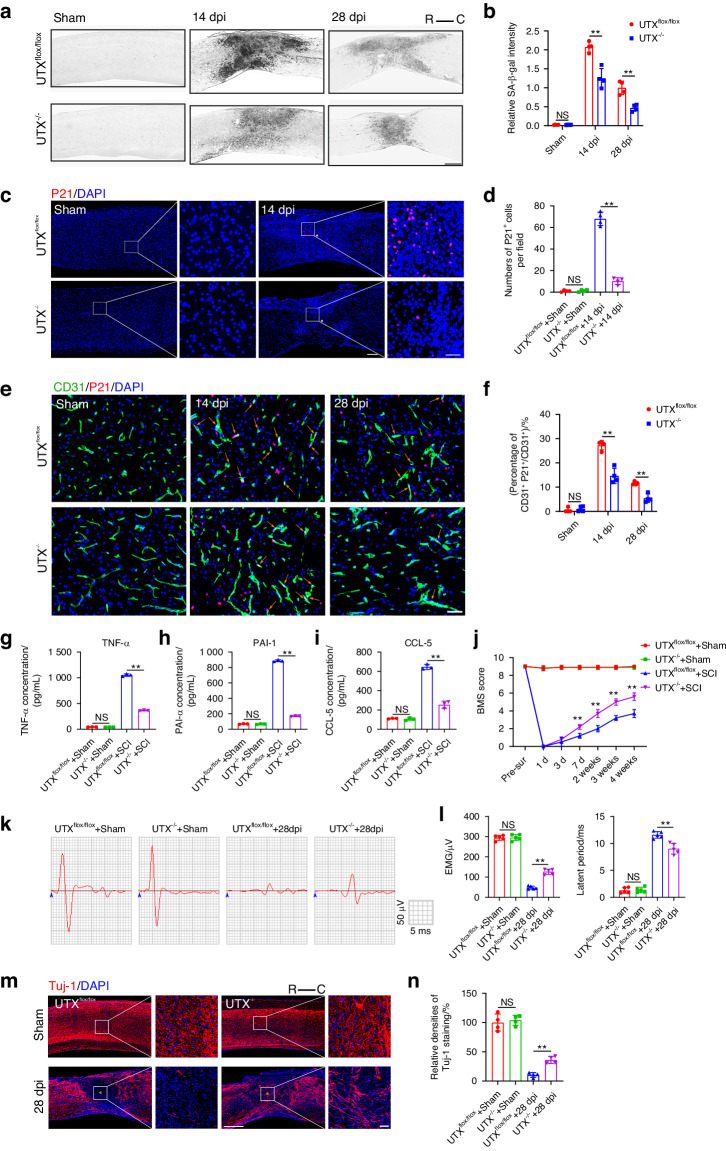
Conditional knockout of UTX in ECs impaired cell senescence and facilitated neurological recovery post SCI. **a** SA-β-gal staining of spinal cord before injury and at 14days, 28days post SCI in UTX^flox/flox^ and UTX^−/−^ mice. Scale bar: 500 μm. R represents rostral, C represents caudal. **b** Quantification of relative SA-β-gal intensity in (**a**) (*n* = 4). **c** Representative images of P21 staining before injury and 14 days after SCI in UTX^flox/flox^ and UTX^−/−^ mice. Scale bar: 200 μm. Scale bar for magnified images: 50 μm. The asterisk indicates the lesion center. **d** Quantification of number of P21^+^ cells in (**c**) (*n* = 4). **e** Representative images of CD31 (green) and P21 (red) staining before injury and at 14 days, 28 days post SCI in UTX^flox/flox^ and UTX^/^mice. Scale bar: 50 μm. The orange arrow indicates CD31^+^P21^+^ cell. **f** Quantification of percentages of CD31^+^P21^+^/CD31^+^ cells in (**e**) (*n* = 4). **g** Elisa test for TNF-α expression in before injury and 14days after SCI in UTX^flox/flox^ and UTX^−/−^ mice (*n* = 3). **h** Elisa test for PAI-1 expression in before injury and 14 days after SCI in UTX^flox/flox^ and UTX^−/−^ mice (*n* = 3). **i** Elisa test for CCL-5 expression in before injury and 14days after SCI in UTX^flox/flox^ and UTX^−/−^ mice (*n* = 3). **j** BMS scores at different time points of UTX^flox/flox^ and UTX^−/−^ mice (*n* = 5). **k** Neuroelectrophysiology analysis of UTX^flox/flox^ and UTX^−/−^ mice before injury and 28 days after SCI. **l** Quantification of motor evoked potential and latent period in (**k**) (*n* = 5). **m** Representative images of Tuj-1 staining of UTX^flox/flox^ and UTX^−/−^ mice before injury and 28 days after SCI. Scale bar: 500 μm. Scale bar for magnified images: 50 μm. The asterisk indicates the lesion center. **n** Quantification of relative Tuj-1 densities in (**m**) (*n* = 4). Data are presented as the mean ± SD. **P < 0.01, ns not significant

After observing an increased expression of UTX in SCMECs following SCI, we aimed to investigate whether EC senescence was intrinsically regulated by UTX and contributed to spinal cord regeneration. To address this, we generated a transgenic mouse model with ECs conditionally deleted of UTX by crossbreeding Tek-Cre mice with UTX^flox/flox^ mice (Fig. S4a). Genotyping confirmed the presence of Tek-Cre and UTX^flox/flox^ alleles in the respective mice (Fig. S4b). Immunofluorescence staining of CD31 and UTX in spinal cord tissues validated the successful generation of ECs UTX^−/−^ mice (Fig. S4c). To explore the effect of UTX on endothelial cell senescence, we extracted BMECs from UTX^−/−^ mice and UTX^flox/flox^ mice, and intervened with H_2_O_2_. Compared to the UTX^flox/flox^ group, the proportions of P53^+^ and P21^+^ BMECs extracted from UTX^−/−^ mice were significantly reduced (Fig. S5). Analyzing common senescence markers upon SCI, UTX^−/−^ mice exhibited fewer senescent cells compared to UTX^flox/flox^ mice, as evidenced by SA-β-gal and p21 staining (Fig. 4a–d). Further exploration of the effect of UTX deletion on SCMECs senescence revealed a significant reduction in CD31^+^P21^+^ and CD31^+^P53^+^ cells in UTX^−/−^ mice compared to UTX^flox/flox^ mice after SCI (Fig. 4e, f, Fig. S6). Since senescent cells secrete SASP, we assessed the effect of UTX knockout on the expression of representative SASP factors using ELISA. The results showed that the expressions of SASP factors such as TNF-α, PAI-1, and CCL-5 were significantly suppressed in UTX^−/−^ mice compared to UTX^flox/flox^ mice after SCI, suggesting that deletion of UTX in ECs alleviated the proinflammatory microenvironment (Fig. 4g–i). To evaluate the effect of UTX deletion on neurological functional recovery after SCI, locomotor behavior tests and electrophysiological evaluations were performed. UTX^−/−^ mice exhibited higher basso mouse scale (BMS) scores and improved electrophysiological parameters compared to UTX^flox/flox^ mice at corresponding time points post-SCI, indicating the importance of UTX in mediating neurological functional repair (Fig. 4j–l). Axon regeneration, a key factor in neurological recovery after SCI, was significantly enhanced in UTX^−/−^ mice compared to UTX^flox/flox^ mice (Fig. 4m, n). Bladder function assessments showed that UTX knockout reduced cell senescence and improved neurological functional recovery after SCI (Fig. S7a, b). These results collectively suggest that UTX plays a crucial role in regulating EC senescence in the injured spinal cord, influencing the proinflammatory microenvironment and contributing to improved neurological recovery after SCI.

### Knockdown of UTX in endothelial cells impaired cell senescence and promoted endothelial cell activity in vitro

**Fig. 5:**
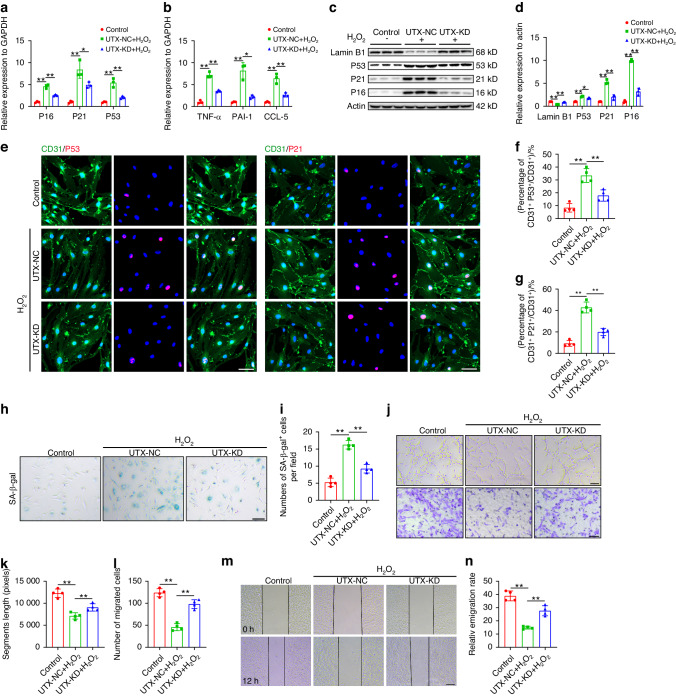
Knockdown of UTX in endothelial cells impaired cell senescence and promoted endothelial cell activity in vitro. **a** Relative expression of P16, P21 and P53 in control, UTX-NC and UTX-KD treated bEnd.3 cells by qRT-PCR (*n* = 3). **b** Relative expression of TNF-α, PAI-1 and CCL-5 in control, UTX-NC and UTX-KD treated bEnd.3 cells by qRT-PCR (*n* = 3). **c** Western blot analysis of Lamin B1, P16, P21 and P53 expression in control, UTX-NC and UTX-KD treated bEnd.3 cells. **d** Quantification of LaminB1, P16, P21 and P53 expression in (**c**) (*n* = 3). **e** Representative images of CD31 (green) and P53 (red), CD31 (green) and P21 (red) in control, UTX-NC and UTX-KD treated bEnd.3 cells. Scale bar: 20 μm. **f** Quantification of percentages of CD31^+^P53^+^/CD31^+^ cells in (**e**) (*n* = 4). **g** Quantification of percentages of CD31^+^P21^+^/CD31^+^ cells in (**e**) (*n* = 4). **h** Representative images of SA-β-gal staining in control, UTX-NC and UTX-KD treated bEnd.3 cells. Scale bar: 100 μm. **i** Quantification of SA-β-gal^+^ cells in (**h**) (*n* = 4). **j** Representative images of bEnd.3 canaliculization (upper panel) and transwell migration (lower panel) in control, UTX-NC and UTX-KD treated bEnd.3 cells. Scale bar: 100 μm. **k** Quantification of segments lengths in (**j**) (*n* = 4). **l** Quantification of migrated cells in (**j**) (*n* = 4). **m** Representative images of the horizontal migration in control, UTX-NC and UTX-KD treated bEnd.3 cells. Scale bar: 200 μm. **n** Quantification of relative migration rate in (**m**) (*n* = 4). Data are presented as the mean ± SD. *P < 0.05, **P < 0.01

To validate our in vivo findings and further elucidate the impact of UTX on endothelial cell senescence, we generated UTX knockdown lentivirus (UTX-KD) and control virus (UTX-NC) for transfection into bEnd.3 cells, followed by treatment with H_2_O_2_. The efficiency of UTX knockdown in bEnd.3 cells was confirmed through immunofluorescence, qRT-PCR, and western blotting, demonstrating a significant reduction in UTX expression (Fig. S8). Subsequent analyses focused on senescent markers (P16, P21, P53) and SASP factors (TNF-α, PAI-1, CCL-5) in different intervention groups. H_2_O_2_ treatment increased the expression of senescent markers and SASP factors, while UTX knockdown resulted in a decrease in their expression levels (Fig. 5a, b). Western blot results further supported this trend, showing that UTX knockout mitigated the upregulation of P53, P21, and P16 proteins induced by H_2_O_2_, consistent with mRNA levels. Conversely, Lamin B1 protein expression exhibited an opposite trend (Fig. 5c, d). Immunofluorescence analysis of P53 and P21 in bEnd.3 cells revealed an increase in P53^+^ and P21^+^ cells after H_2_O_2_ treatment, which significantly decreased upon UTX knockdown (Fig. 5e–g). SA-β-gal staining confirmed these observations, showing a higher number of senescent cells after H_2_O_2_ treatment in UTX-NC groups, while UTX knockdown substantially reduced the number of SA-β-gal^+^ cells (Fig. 4h, i).

Functional assessments demonstrated that UTX regulates endothelial cell senescence in response to H_2_O_2_ and modulates the biological function of bEnd.3 cells. Segment length, the number of migrated cells, and horizontal migration distance significantly increased in UTX-KO groups compared to UTX-NC groups after H_2_O_2_ treatment (Fig. 5j–n). These results underscore the role of UTX in governing ECs senescence and functional outcomes in the context of oxidative stress.

### UTX regulated endothelial cell senescence via directly targeting CNN1

**Fig. 6:**
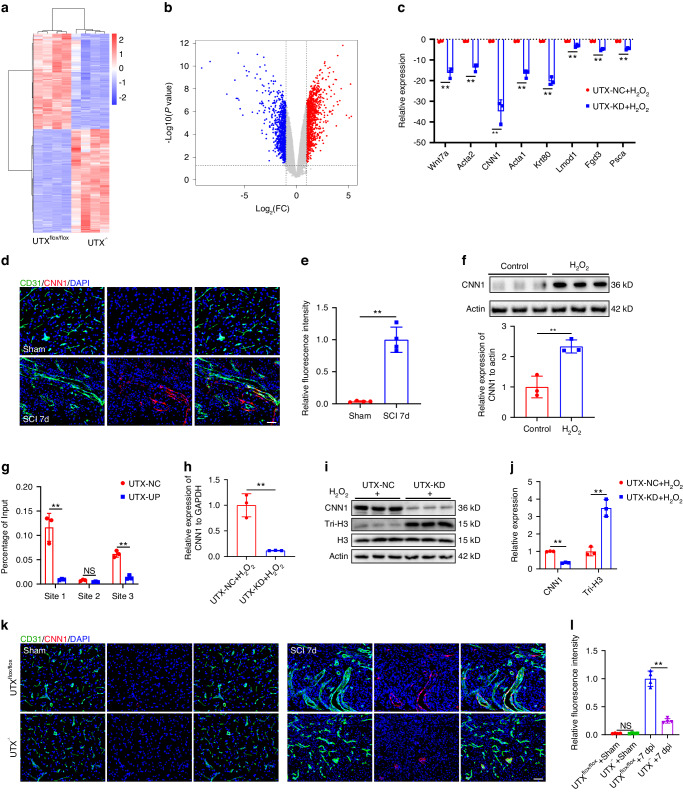
UTX regulated endothelial cell senescence via directly targeting CNN1. **a** Differentially expressed genes of BMECs extracted from UTX^flox/flox^ mice and UTX^−/−^ mice after H_2_O_2_ treatment (*n* = 4). **b** Volcanic map showing the differential expression of BMECs from UTX^flox/flox^ mice and UTX^−/−^ mice (*n* = 4). Gray dots represent genes that are not differentially expressed, blue dots represent down-regulated genes, and red dots represent up-regulated genes. **c** Verification of top 8 down-regulated genes by qRT-PCR (*n* = 3). **d** Representative images of CD31 (Green) and CNN1 (Red) staining in sham and SCI 7days group. Scale bar: 50 μm. **e** Quantification of relative fluorescence intensity of CNN1 in (**d**) (*n* = 4). **f** Relative expression and quantification of CNN1 in CNN1-NC and CNN1-OE treated bEnd.3 cells by WB (*n* = 3). **g** ChIP-qPCR analysis of targeting binding of UTX and the H3K27me3 in the promoter region of CNN1 (*n* = 3). **h** Relative expression of CNN1 in UTX-NC and UTX-KD bEnd.3 cells under H_2_O_2_ treatment by qRT-PCR (*n* = 3). **i** Western blot analysis of CNN1 and tri-histone in UTX-NC and UTX-KD bEnd.3 cells under H_2_O_2_ treatment. **j** Quantification of CNN1 and tri-histone expression in (**i**) (*n* = 3). **k** Representative images of CD31 (green) and CNN1 (red) staining before injury and at 28 days post SCI in UTX^flox/flox^ and UTX^−/−^ mice. Scale bar: 50 μm. **l** Quantification of relative fluorescence intensity of CNN1 in (**k**) (*n* = 4). Data are presented as the mean ± SD. **P < 0.01, ns not significant

To unravel the molecular mechanisms governing the regulation of endothelial cell senescence by UTX, we isolated primary BMECs from UTX^flox/flox^ mice and UTX^−/−^ mice, induced cellular senescence with H_2_O_2_, and conducted RNA-seq to identify downstream target genes of UTX (Fig. 6a). Analysis revealed 1 949 differentially expressed genes, with 1 013 up-regulated and 936 down-regulated genes (Fig. 6b). The top 8 down-regulated genes, including calponin 1 (CNN1), were verified by qRT-PCR, showing a significant downregulation of CNN1 upon UTX knockdown in ECs subjected to H_2_O_2_ interventions (Fig. 6c). Immunofluorescence staining demonstrated low CNN1 expression in normal spinal cord tissue, significantly increased on day 7 post-SCI, primarily in or around endothelial cells (Fig. 6d, e). Senescent bEnd.3 cells induced by H_2_O_2_ exhibited higher CNN1 expression (Fig. 6f).

To explore the role of elevated CNN1 expression in endothelial cell senescence, we generated CNN1 overexpressing lentivirus (CNN1-OE) and transfected it into bEnd.3 cells. CNN1 overexpression up-regulated CNN1 expression at both the gene and protein levels and increased the expression of senescent markers and SASP factors (Fig. S9a–d). CNN1 overexpression induced cell senescence, characterized by an increased number of SA-β-gal^+^ cells, and decreased segments length and migrated cells compared to the control group (Fig. S9e–j). ChIP-qPCR assays were conducted to investigate the targeted binding relationships between UTX and CNN1. Three primers targeting potential binding sites of CNN1 at its promoter region were designed, revealing differential expression at sites 1 and 3, indicating UTX regulation of CNN1 expression through histone demethylation (Fig. 6g). Knockdown of UTX significantly reduced the increase in CNN1 expression caused by H_2_O_2_ intervention, and WB confirmed the downregulation of CNN1 expression after UTX knockdown, suggesting that UTX may affect CNN1 expression through demethylase effect (Fig. 6h–j). Immunofluorescence staining of spinal cord tissue from injured UTX^flox/flox^ mice and UTX^−/−^ mice indicated that UTX deletion effectively reduced CNN1 expression in SCMECs 7 days after injury (Fig. 6k, l). These findings collectively suggest that UTX regulates ECs senescence through the direct targeting of CNN1 via binding to its promoter region.

### Deletion of UTX resulted in a reduction of senescence in SCMECs and contributed to improved neurological recovery through the downregulation of CNN1

**Fig. 7:**
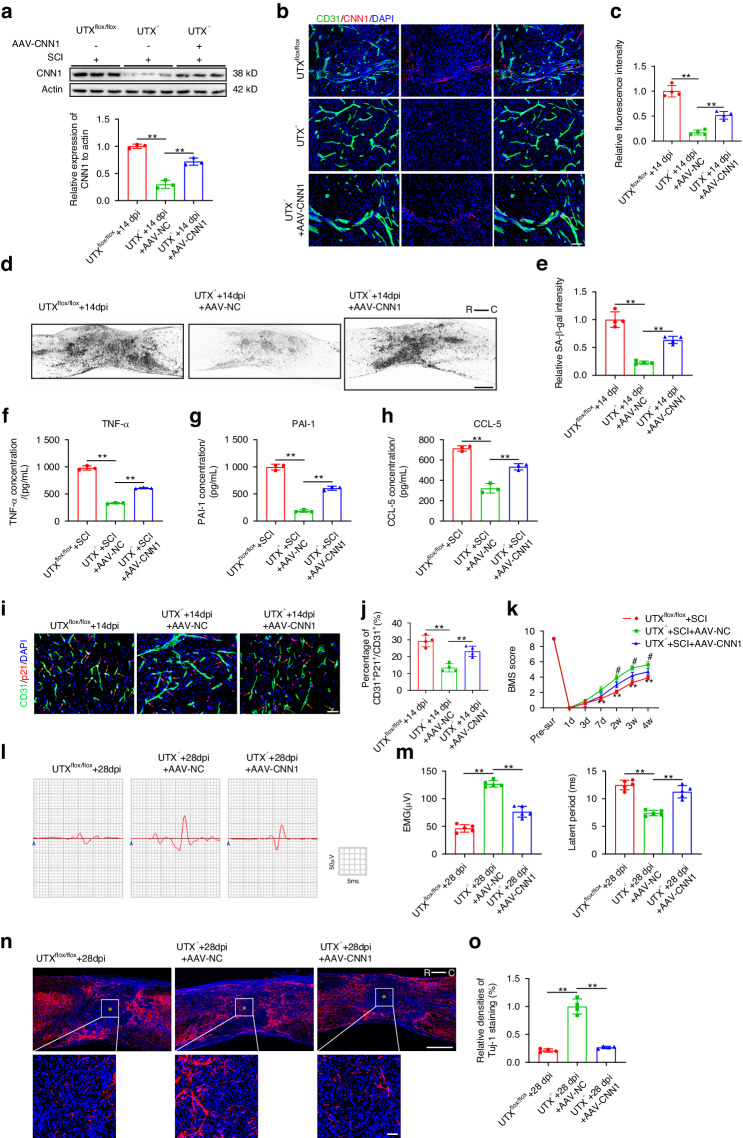
Deletion of UTX in ECs reduced senescence in SCMECs and enhanced neurological recovery by downregulating CNN1. **a** Western blot AND quantification of CNN1 expression in UTX^flox/flox^, UTX^−/−^ + AAV-NC and UTX^−/−^ + AAV-CNN1 groups after SCI. **b** Representative images of CD31 (Green) and CNN1 (Red) staining in UTX^flox/flox^, UTX^−/−^ + AAV-NC and UTX^−/−^ + AAV-CNN1 groups after SCI. Scale bar: 50 μm. **c** Quantification of relative fluorescence intensity of CNN1 in (**b**) (*n* = 4). **d** Representative SA-β-gal images in UTX^flox/flox^, UTX^−/−^ + AAV-NC and UTX^−/−^ + AAV-CNN1 groups after SCI. Scale bar: 500 μm. R represents rostral, C represents caudal. **e** Quantification of relative SA-β-gal intensity in (**d**) (*n* = 4). **f** Elisa test for TNF-α expression in UTX^flox/flox^, UTX^−/−^ + AAV-NC and UTX^−/−^ + AAV-CNN1 groups after SCI (*n* = 3). **g** Elisa test for PAI-1 expression in UTX^flox/flox^, UTX^−/−^ + AAV-NC and UTX^−/−^ + AAV-CNN1 groups after SCI (*n* = 3). **h** Elisa test for CCL-5 expression in UTX^flox/flox^, UTX^−/−^ + AAV-NC and UTX^−/−^ + AAV-CNN1 groups after SCI (*n* = 3). **i** Representative images of CD31 (green) and p21 (red) staining 14days post SCI in UTX^flox/flox^, UTX^−/−^ + AAV-NC and UTX^−/−^ + AAV-CNN1 groups. Scale bar: 50 μm. The orange arrow indicates CD31^+^P21^+^ cell. **j** Quantification of percentages of CD31^+^P21^+^/CD31^+^ cells in (**i**) (*n* = 4). **k** BMS scores at different timepoints in UTX^flox/flox^, UTX^−/−^ + AAV-NC and UTX^−/−^ + AAV-CNN1 groups (*n* = 5). **l** Neuroelectrophysiology analysis of UTX^flox/flox^, UTX^−/−^ + AAV-NC and UTX^−/−^ + AAV-CNN1 mice 28 days after SCI. **m** Quantification of motor evoked potential and latent period in (**l**) (*n* = 5). **n** Representative images of Tuj-1 staining of UTX^flox/flox^, UTX^−/−^ + AAV-NC and UTX^−/−^ + AAV-CNN1 mice 28 days after SCI. Scale bar: 500 μm. Scale bar for magnified images: 50 μm. The asterisk indicates the lesion center. **o** Quantification of relative Tuj-1 densities in (**n**) (*n* = 4). Data are presented as the mean ± SD. **P < 0.01, ^#^*P* < 0.05, ns=not significant

In the investigation of UTX regulation on SCMECs senescence through CNN1, an adeno-associated virus overexpressing CNN1 (AAV-CNN1) was utilized. Local injection of AAV-CNN1 into UTX^−/−^ mice at 7 days post SCI resulted in a significant increase in CNN1 expression compared to UTX^−/−^ mice treated with control AAV (AAV-NC) (Fig. 7a). Immunofluorescence staining confirmed the reversal of CNN1 downregulation by AAV-CNN1 administration in UTX^−/−^ mice (Fig. 7b, c). The exploration of the UTX-CNN1 axis’s impact on ECs senescence revealed increased SA-β-gal intensity and elevated levels of SASP factors (TNF-α, PAI-1, and CCL-5) after AAV-CNN1 injection, partially counteracting the effect of UTX knockout (Fig. 7d–h). Furthermore, AAV-CNN1 administration increased the percentages of P21^+^ SCMECs in injured spinal cord tissue after UTX-specific deletion in ECs, indicating a role in promoting SCMECs senescence (Fig. 7i, j, Fig. S10). Behavioral assessments, electrophysiological analysis, and axon density measurements demonstrated that AAV-CNN1 attenuated the beneficial effects observed in UTX^−/−^ mice, negatively impacting neurological recovery (Fig. 7k–o). Bladder function assessments revealed a reversal of improvements in bladder diameter and detrusor muscle thickness in UTX^−/−^ mice after AAV-CNN1 injection (Fig. S7c, d), further highlighting the crucial role of the UTX-CNN1 epigenetic axis in regulating vascular endothelial cell senescence and influencing neuroinflammation and neurological recovery post-SCI.

### UTX knockdown depressed cell senescence and improved endothelial activity by downregulating CNN1 in vitro

**Fig. 8:**
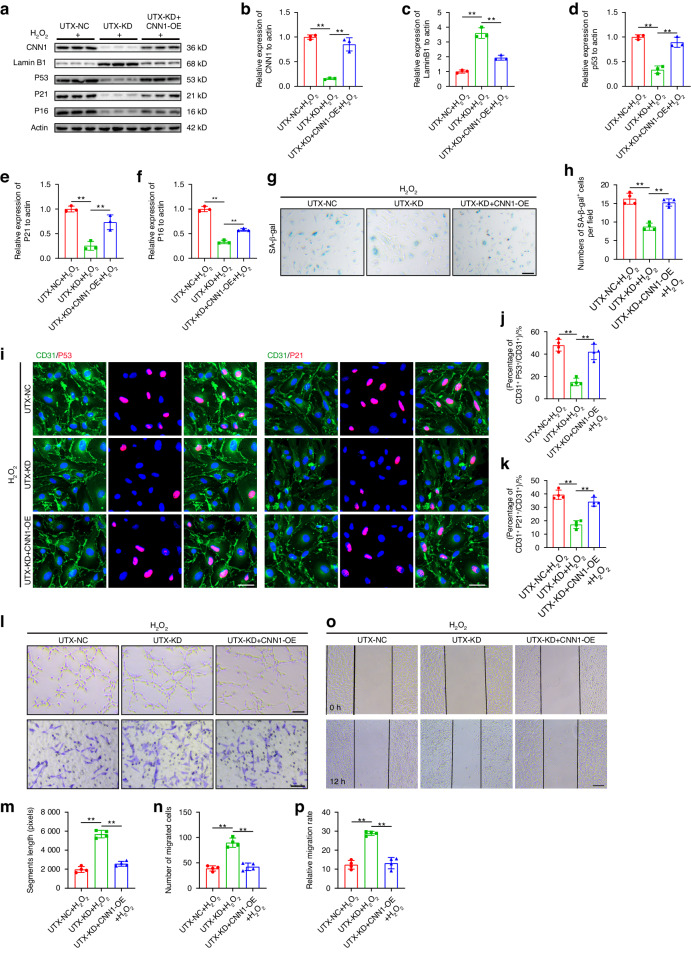
UTX knockdown depressed cell senescence and improved endothelial activity by downregulating CNN1 in vitro. **a** Western blot analysis of CNN1, Lamin B1, P16, P21 and P53 expression in UTX-NC, UTX-KD and UTX-KD + CNN1-OE groups. **b** Quantification of CNN1 expression in (**a**) (*n* = 3). **c** Quantification of LaminB1 expression in (**a**) (*n* = 3). **d** Quantification of P53 expression in (**a**) (*n* = 3). **e** Quantification of P21 expression in (**a**) (*n* = 3). **f** Quantification of p16 expression in (**a**) (*n* = 3). **g** Representative images of SA-β-gal staining in UTX-NC, UTX-KD and UTX-KD + CNN1-OE groups. Scale bar: 100 μm. **h** Quantification of SA-β-gal^+^ cells in (**g**) (*n* = 4). **i** Representative images of CD31 (green) and P53 (red), CD31 (green) and P21 (red) in UTX-NC, UTX-KD and UTX-KD + CNN1-OE groups. Scale bar: 20 μm. **j** Quantification of percentages of CD31^+^P53^+^/CD31^+^ cells in (**i**) (*n* = 4). **k** Quantification of percentages of CD31^+^P21^+^/CD31^+^ cells in (**i**) (*n* = 4). **l** Representative images of bEnd.3 canaliculization (upper panel) and transwell migration (lower panel) in UTX-NC, UTX-KD and UTX-KD + CNN1-OE groups. Scale bar: 100 μm. **m** Quantification of segments lengths in (**l**) (*n* = 4). **n** Quantification of migrated cells in (**l**) (n = 4). **o** Representative images of the horizontal migration in UTX-NC, UTX-KD and UTX-KD + CNN1-OE groups. Scale bar: 200 μm. **p** Quantification of relative migration rate in (**m**) (*n* = 4). Data are presented as the mean ± SD. **P < 0.01

We conducted in vitro experiments to further elucidate the role of the UTX-CNN1 axis in regulating endothelial cell senescence. As shown in Fig. 8a–f, UTX knockdown decreased the expression of CNN1 induced by H_2_O_2_ treatment. Transfection of CNN1 overexpressing lentivirus (CNN1-OE) increased the expression of CNN1 in ECs. Under H_2_O_2_ intervention, UTX knockdown downregulated the expression of senescent markers (P16, P21, and P53) in ECs, while increasing the expression of Lamin B1. CNN1 overexpression reversed these effects, increasing the expression of senescent markers and decreasing Lamin B1 expression. SA-β-gal staining demonstrated that overexpression of CNN1 in ECs attenuated the effect of UTX knockdown on decreasing the number of SA-β-gal^+^ cells (Fig. 8g, h). Immunofluorescence results showed that under H_2_O_2_ intervention, UTX knockdown decreased the percentages of P21^+^ and P53^+^ cells. However, upregulation of CNN1 rescued the function of H_2_O_2_ in inducing cell senescence, as evidenced by increased P21^+^ and P53^+^ cell percentages (Fig. 8i–k). We further investigated whether the UTX-CNN1 axis could affect the biological activity of endothelial cells. UTX knockdown in ECs, under H_2_O_2_ treatment, increased vascular segment length and the number of migrated cells compared to the UTX negative control. However, CNN1 overexpression attenuated these effects (Fig. 8l–n). UTX knockdown also promoted migration distance, and CNN1 overexpression attenuated this effect (Fig. 8o, p). These results collectively suggest that the UTX-CNN1 axis plays a crucial role in regulating endothelial cell senescence and influencing cellular biological activity, with UTX knockdown leading to reduced senescence and enhanced cell activity, while CNN1 overexpression counteracts these effects.

### Senolytic drug enhanced functional healing via reducing endothelial cell senescence and inhibiting release of SASP factors

**Fig. 9:**
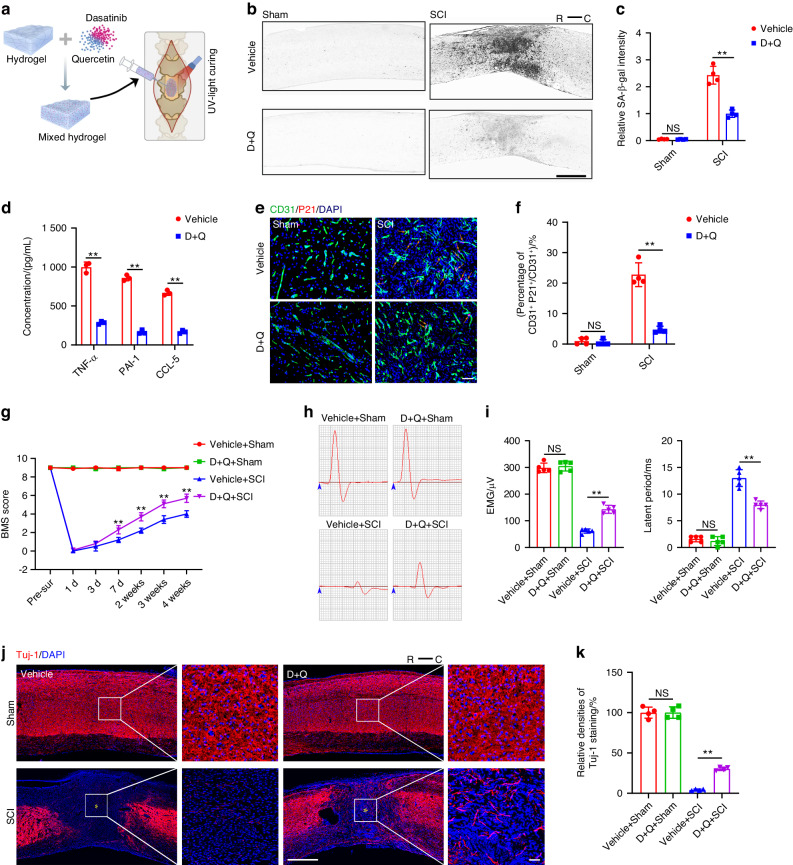
Senolytic drugs improved functional recovery by reducing endothelial cell senescence and inhibiting the release of SASP factors. **a** Schematic diagram of local senolytic drugs administration. **b** Representative SA-β-gal images in vehicle and D + Q treated groups before and after SCI. Scale bar: 500 μm. R represents rostral, C represents caudal. **c** Quantification of relative SA-β-gal intensity in (**b**) (*n* = 4). **d** Elisa test for TNF-α, PAI-1 and CCL-5 expression in vehicle and D + Q treated groups after SCI (*n* = 3). **e** Representative images of CD31 (green) and P21 (red) staining before and after SCI in vehicle and D + Q treated mice. Scale bar: 50 μm. The orange arrow indicates CD31^+^P21^+^ cell. **f** Quantification of percentages of CD31^+^P21^+^/CD31^+^ cells in (**e**) (*n* = 4). **g** BMS scores at different time points of vehicle and D + Q treated mice (*n* = 5). **h** Neuroelectrophysiology analysis of vehicle and D + Q treated mice before injury and 28 days after SCI. **i** Quantification of motor evoked potential and latent period in (**h**) (*n* = 5). **j** Representative images of Tuj-1 staining of vehicle and D + Q treated mice before injury and 28 days after SCI. Scale bar: 500 μm. Scale bar for magnified images: 50 μm. The asterisk indicates the lesion center. **k** Quantification of relative Tuj-1 densities in (**j**) (*n* = 4). Data are presented as the mean ± SD. **P < 0.01, ns=not significant

In the context of senescent cell accumulation impeding neurological functional recovery post SCI, we investigated the potential therapeutic effects of senolytic drugs, specifically dasatinib and quercetin (D + Q), on neurological functional recovery. Utilizing a slow drug release system involving a hydrogel to maximize drug effectiveness (Fig. 9a), we observed that D + Q treatment had no significant influence on SA-β-gal intensity in the normal spinal cord. However, following SCI, D + Q administration noticeably attenuated SA-β-gal intensity compared to the control groups (Fig. 9b, c). Furthermore, D + Q treatment decreased the expression levels of SASP factors, including TNF-α, PAI-1, and CCL-5, in the injured spinal cord tissue (Fig. 9d). Immunofluorescence results revealed a marked reduction in P21^+^CD31^+^ cells and P53^+^CD31^+^ cells after D + Q treatment compared to the control group (Fig. 9e, f, Fig. S11a, b). The data indicated that local administration of D + Q could suppress cellular senescence and alleviate the local proinflammatory microenvironment. A preliminary validation of the impact of D + Q treatment on the expression of the epigenetic regulatory factor UTX showed a significant reduction in UTX expression in SCMECs following D + Q treatment (Fig. S11c-d). BMS scores indicated that mice treated with D + Q exhibited significantly higher scores from 7 days to 4 weeks post-SCI, suggesting improved functional recovery compared with the vehicle groups (Fig. 9g). Electrophysiological analysis demonstrated that D + Q treatment improved neural conductive capacity, as indicated by decreased latency and increased evoked potential compared to the vehicle group (Fig. 9h, i). Tuj-1 staining revealed that D + Q treatment increased the density of axon fibers in the injured spinal cord tissue at 28 days post-SCI (Fig. 9j, k).

**Fig. 10:**
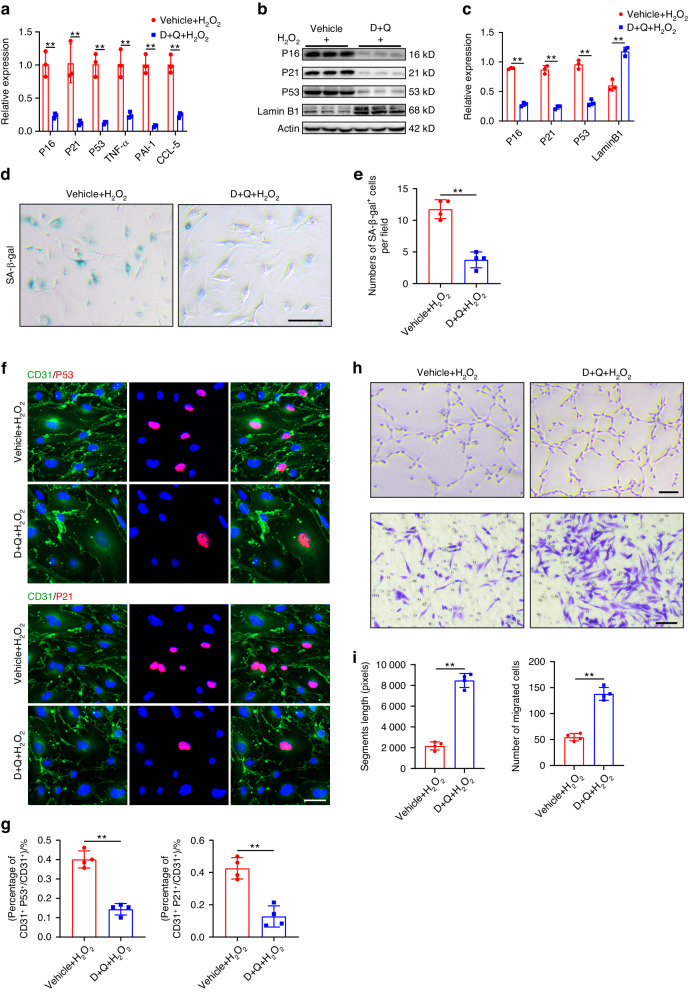
D + Q treatment decreased H2O2-induced senescence in endothelial cells and enhanced endothelial activity in vitro. **a** Relative expression of senescent markers and SASP factors detected by qRT-PCR in vehicle and D + Q treated bEnd.3 cells (*n* = 3). **b** Western blot analysis of Lamin B1, P16, P21 and P53 expression in vehicle and D + Q treated bEnd.3 cells. **c** Quantification of relative expression of Lamin B1, P16, P21 and P53 in (**b**) (*n* = 3). **d** SA-β-gal staining of bEnd.3 cells under H_2_O_2_ treatment in vehicle and D + Q treated groups. Scale bar: 100 μm. **e** Quantification of SA-β-gal^+^ cells in (**d**) (*n* = 4). **f** Representative images of CD31 (green) and P53 (red), CD31 (green) and P21 (red) in vehicle and D + Q treated groups. Scale bar: 20 μm. **g** Quantification of percentages of CD31^+^P53^+^/CD31^+^ cells and CD31^+^P21^+^/CD31^+^ cells in (**f**) (*n* = 4). **h** Representative images of bEnd.3 canaliculization (upper panel) and transwell migration (lower panel) in vehicle and D + Q treated groups. Scale bar: 100 μm. **i** Quantification of segments lengths and migrated cells in (**h**) (*n* = 4). Data are presented as the mean ± SD. **P < 0.01

In vitro experiments showed that D + Q treatment down-regulated the mRNA expression of senescent markers (P16, P21, P53) and SASP factors (TNF-α, PAI-1, CCL-5) in ECs. D + Q treatment rescued the senescence phenotype induced by H_2_O_2_, evidenced by reduced SA-β-gal^+^, P53^+^, and P21^+^ ECs (Fig. 10a–c). SA-β-gal staining and immunofluorescent staining results demonstrated that D + Q treatment attenuated the effect of H_2_O_2_ on inducing endothelial cell senescence, with reduced SA-β-gal^+^, P53^+^, and P21^+^ ECs compared to vehicle-treated ECs (Fig. 10d–g). Additionally, D + Q administration increased vascular segment length and the number of migrated cells, indicating a positive impact on the biological activity of endothelial cells, particularly under H_2_O_2_ treatment (Fig. 10h, i). These results collectively suggest that local administration of D + Q has strong senolytic activity, reducing senescent ECs and inhibiting the accumulation of SASP factors. This creates a regenerative microenvironment that may contribute to functional recovery after SCI.

## Discussion

**Fig. 11:**
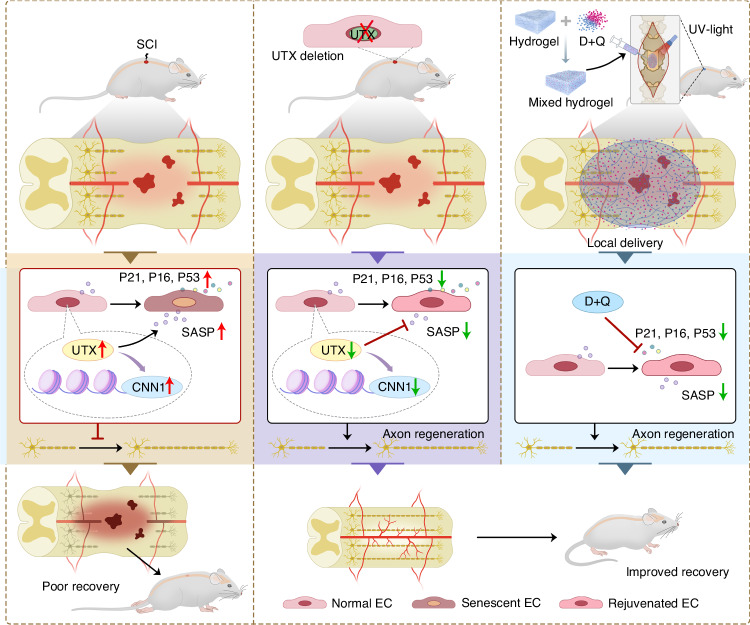
A schematic of UTX/CNN1 axis in the regulation of SCMECs senescence. Acute spinal cord injury (SCI) triggered SCMECs senescence. Mechanistically, the elevated UTX expression interacted with CNN1 to form an epigenetic regulatory axis, which further mediates SCMECs senescence, SASP secretion, neuroinflammation and neurological recovery. Local administration of dasatinib and quercetin reduced SCMECs senescence and levels of proinflammatory factors, finally restoring the local regenerative microenvironment promoting functional recovery after SCI

SCI is a severe condition arising from traumatic damage to the spinal cord, resulting in a variety of physical and neurological impairments. The current dearth of effective treatments can be ascribed to the inadequate exploration of the pathophysiological changes that occur after SCI.^28^ In this study, we initially characterized senescence in SCMECs as a responsive phenomenon to acute SCI. The observed upregulation of UTX after SCI demonstrated a discernible correlation with SCMECs senescence, concurrently exacerbating the SASP secretion and intensifying neuroinflammation. In contrast, the conditional deletion of UTX in endothelial cells imparted protection against SCMECs senescence, alleviated the release of proinflammatory SASP factors, optimized the local microenvironment, and facilitated neurological functional recovery post-SCI. Mechanistically, a direct interaction between UTX and calponin 1 (CNN1) was elucidated, establishing an epigenetic regulatory axis. This UTX-CNN1 axis played a pivotal role in mediating trauma-induced SCMECs senescence, SASP secretion, neuroinflammation, and subsequent neurological functional repair. The local administration of the senolytic combination of dasatinib and quercetin (D + Q) resulted in reduced UTX expression in SCMECs, attenuated senescence, diminished proinflammatory factor secretion, and consequently restored the local regenerative microenvironment. These findings suggest potential innovative therapeutic targets for SCI treatment (Fig. 11).

Numerous intrinsic and extrinsic factors can instigate cellular senescence, encompassing DNA damage, oxidative stress, mitochondrial dysregulation, and telomere shortening.^29,30^ Senescent cells manifest increased expression of senescence-related markers, such as SA-β-gal, P53, P21, and P16. Moreover, they release a spectrum of SASP factors, including proinflammatory cytokines, chemokines, various growth factors, and proteases. This combined secretion contributes to the modulation of the local microenvironment.^10,31^ Senescent cells possess the potential to establish a proinflammatory environment, thereby contributing to the progression of aging neuroinflammatory disorders.^32^ Nevertheless, the role, characteristics, and mechanisms governing trauma-induced cellular senescence remain incompletely understood. Mounting evidence suggests that the physiological significance of cellular senescence extends beyond aging diseases to encompass crucial biological processes such as embryonic development, wound healing, and tissue repair.^33,34^ The multifunctional nature of cellular senescence raises the question of whether the fundamental features of cell senescence underlying these diverse biological processes may differ. Importantly, SASP factors exhibit distinct features in different cell types and under different senescence-inducing stressors.^13^ Young et al. demonstrated that multiple cells exhibiting senescent characteristics are “transiently” present during muscle regeneration.^35^ Emerging research has also identified DNA damage-induced cellular senescence as a potential driver of traumatic brain injury-related sequelae.^36^ In our current study, we observed that SCI could induce senescence in spinal cord cells, as indicated by the expression of SA-β-gal, P16, P21, and P53, along with lower expression of Lamin B1. Our data revealed that a significant proportion of senescent cells in the injured spinal cord are SCMECs. Furthermore, our investigation revealed that senescent endothelial cells exhibit impaired biological functions and display distinctive features within the SASP, including heightened expression of TNF-α, PAI-1, and CCL-5, as elucidated by PCR-array analysis. Plasminogen activator inhibitor 1 (PAI-1), as a common senescent marker and SASP factor has been identify up regulated in plasma and brain of patients with Alzheimer’s disease, which might contribute to the progress of Alzheimer’s disease by inducing cell senescence.^37^ Tumor necrosis factor-alpha (TNF-α), an important pro-inflammatory cytokine secreted by some types of senescent cells, can induce senescence in mouse and human cells.^38^ C–C motif chemokine ligand (CCL-5) is a chemokine recognized for its role in promoting chemotactic signaling to T lymphocytes, basophils, and eosinophils in the peripheral immune system. Elevated levels of CCL-5 have been linked to Alzheimer’s disease (AD) in patients.^39^ These pieces of evidence suggest that senescent SCMECs, also referred to as “zombie cells,” resulting from traumatic SCI, may secrete multiple pro-inflammatory SASP factors, creating an unfavorable microenvironment and leading to poor functional recovery after SCI. Trauma-induced endothelial cell (EC) senescence, as investigated in our study, exhibits several distinctive features when compared to the natural aging process of ECs. While both processes involve alterations in cellular behavior and molecular signaling, the origins and implications differ significantly. Initiated by acute trauma, traumatic-induced EC senescence is driven by the sudden and intense stressors associated with SCI. DNA damage, oxidative stress, and inflammatory cascades play pivotal roles in this context. Aging-related EC senescence, on the other hand, evolves gradually over time and is influenced by intrinsic and extrinsic factors associated with the aging process.^40^ Upon further investigation into the molecular signature governing senescent ECs in the injured spinal cord and their role in the process of SCI healing, genetic manipulation or elimination of senescent cells emerges as a more promising avenue. This may pave the way for novel therapeutic strategies in SCI repair.

Epigenetic modifications, encompassing DNA methylation, histone modification, and non-coding RNA, have been acknowledged as pivotal drivers of cell senescence.^41^ Significantly, changes in DNA methylation are often associated with a decrease in cell number and self-renewal ability, resembling observations in the aging process. The cellular senescence process entails alterations in the landscapes of DNA methylation, and different cell types manifest distinct gene expression patterns.^42^ Histone modification represents an additional epigenetic regulatory layer that occurs in stress-induced premature senescent cells.^43^ The histone methylase EZH2 inhibits Foxo1 expression, thereby suppressing the antioxidant capacity of BMSCs, ultimately resulting in senescence and osteogenic defects in BMSCs.^18^ In our present study, we initiated the characterization of the epigenetic modification landscape within vascular endothelial cells undergoing senescence after SCI. The expressions of KDM6A (UTX), lysine acetyltransferase 5 (KAT5), suppressor of variegation 3-9 homolog 1 (SUV39H1), and histone deacetylase 1 (HDAC1) were significantly increased, while the expressions of histone deacetylase 3 (HDAC3), methyl CpG binding domain protein 2 (MBD2), and protein arginine methyltransferase 3 (PRMT3) were decreased. These findings suggest that epigenetic modifications play a role in the senescence of vascular endothelial cells during traumatic SCI. Notably, among all the altered epigenetic factors, KDM6A, also known as UTX exhibited significant changes, primarily functioning by removing the trimethyl group from lysine 27 of histone H3 (H3K27me3).^44^ UTX has been reported to regulate cellular senescence in non-traumatic diseases. Deletion of UTX inhibits the cellular senescence pathway and plays a crucial role in chondrocyte homeostasis.^45^ Stem cell culture medium induced the production of fibroblast senescent markers such as SA-β-gal and senescence-associated heterochromatic foci (SAHF), while UTX knockout significantly reduced the expression of SA-β-gal and SASP factors IL-8 and IL-3.^46^ In hematopoietic stem cells, UTX was closely related to the expression of senescent markers, reactive oxygen accumulation, and DNA damage repair.^21^ The H3K27 histone demethylase UTX was also found to be a direct sensor of oxygen, controlling cell fate.^47^ In our present study, we observed high expression of UTX in SCMECs after SCI, with peak expression at 7 days post-injury. We hypothesize that hypoxia resulting from SCI promotes histone methylation, leading to increased UTX expression in endothelial cells (ECs). This heightened UTX expression in ECs may serve as a response to sense oxygen levels at the injury site, subsequently triggering endothelial cell senescence and the secretion of proinflammatory SASP factors after SCI. However, the specific mechanism behind this observation requires further investigation. We then conducted RNA-seq and ChIP-qPCR to identify the downstream target gene of UTX involved in regulating EC senescence. The results suggest that calponin may be a potential downstream target gene regulated by UTX in the context of endothelial cell senescence. Calponin, also known as CNN1, is an actin-associated regulatory protein has its specific function as myosin ATPase.^48,49^ The conventional viewpoint suggests that CNN1 was primarily expressed at high levels during the development of smooth muscle cells. Its role was thought to involve the regulation of smooth muscle differentiation and the enhancement of contractile function.^50,51^ CNN1 was also found to be closely associated with vascular remodeling in hypertension. Matrix metalloproteinase 2 was identified as a down-regulator of CNN1 expression through a posttranslational mechanism. This led to increased proliferation of vascular smooth muscle cells, contributing to chronic arterial remodeling.^50^ CNN1 further activates the ROCK1/MLC pathway, mediating the contraction of tumor-associated fibroblasts. This activation promotes resistance in gastric cancer cells against 5-fluorouracil chemotherapy.^52^ However, the role of CNN1 in CNS diseases and whether CNN1 regulates cell senescence are still rarely investigated. In our research, we discovered for the first time that CNN1 is highly expressed in SCMECs after SCI. CNN1 up-regulates the expression of senescent markers SA-β-gal, P53, P21, P16, and SASP factors, impairing the biological functions of endothelial cells. Deletion of UTX in ECs decreased CNN1 expression by increasing the level of histone methylation in the CNN1 promoter region, thereby reducing SCMECs senescence, repressing SASP secretion, and improving neurological function recovery after SCI. We also observed the features of enhanced axon regeneration following UTX deletion in ECs. We propose that the KDM6A/UTX-CNN1 axis may influence endothelial cell senescence, triggering the release of SASP factors, regulating the local microenvironment, and potentially contributing to the observed improvement in axonal regeneration.

The combination of dasatinib and quercetin (D + Q) is defined as a first generation of senolytic drugs. D + Q treatment has been proven to selectively target senescent cells and attenuate SASP in multiple tissues, which has been demonstrated to be therapeutic for many age-related diseases, such as disc degeneration and atherosclerosis.^53,54^ Diogo et al. previously illustrated the favorable outcomes of senolytic drugs in enhancing functional recovery post-injury by promoting myelin preservation, reducing fibrotic scarring, and mitigating inflammation.^22^ In conjunction with the genetic deletion of UTX in endothelial cells for the regulation of trauma-induced cellular senescence within the injured spinal cord, our investigation comprehensively explores the therapeutic impacts and underlying mechanisms of dasatinib and quercetin (D + Q) in the context of SCI and subsequent repair. We observed that localized administration of D + Q significantly diminished the expression of the epigenetic factor UTX in senescent endothelial cells, lowered the count of senescent endothelial cells, mitigated the secretion of SASP, enhanced the local microenvironment, maintained neuroinflammation equilibrium, and facilitated axon regeneration, ultimately contributing to neurological functional recovery. This study significantly advances our comprehension of the roles and mechanisms of senolytic drugs in central nervous system trauma diseases, underscoring the imperative for further exploration into the diverse biological functions and underlying mechanisms associated with these drugs. Our in vivo outcomes additionally highlight the superior efficacy of hydrogel-based locally sustained D + Q release in comparison to systemic administration, considering the challenges presented by the blood-spinal barrier. Collectively, these findings substantiate D + Q as a promising therapeutic intervention for spinal cord injury treatment.

This study is subject to several limitations. Firstly, our investigation primarily delved into the regulatory impact of UTX on senescence in SCMECs. However, we did not extend our exploration to assess whether UTX influences other cellular processes such as apoptosis, pyroptosis, and ferroptosis in SCMECs. Secondly, while acknowledging the pivotal role of vascular endothelial cells in regeneration and repair, we recognize that the effects of senolytic drugs are multifaceted. These drugs may exert their actions through various mechanisms beyond the clearance of senescent vascular endothelial cells, potentially influencing other types of senescent cells. This emphasizes the complexity of senolytic drug effects and underscores the necessity for further investigation into their precise mechanisms of action. Third, we encountered challenges in obtaining enough primary SCMECs for experimentation. Consequently, we chose to utilize primary brain microvascular endothelial cells as an alternative due to their greater accessibility and more conducive conditions for experimentation.

In summary, our study unveils, for the first time, that SCMECs senescence can be triggered by acute trauma, playing a role in the pathophysiological changes following SCI. Mechanistically, the significantly elevated expression of UTX in injured SCMECs forms an epigenetic regulatory axis by binding to calponin 1 (CNN1). This axis mediates trauma-induced vascular endothelial cell senescence, SASP secretion, neuroinflammation, and promotes spinal cord neurological functional repair. Local administration of the senolytic combination of dasatinib and quercetin reduces the number of senescent SCMECs and the levels of proinflammatory factors, restoring the local regenerative microenvironment and aiding spinal cord functional repair. Targeting this epigenetic regulation mechanism (UTX-CNN1) axis may present a novel treatment strategy for SCI repair.

## Materials and methods

### Animals

Female C57BL/6 mice (8 weeks old, 20–23 g) were purchased from SJA Laboratory Animal Co., Ltd (Hunan, China). UTX^flox/flox^ and Tek-Cre: UTX^flox/flox^ (UTX^−/−^) female mice were obtained by hybridization between Tek-Cre male mice and UTX^flox/flox^ female mice, which were purchased from Jackson Lab, the United States. All animals were kept in the laboratory animal department at Central South University in Changsha, China, and had free access to food and water. All experiments were conducted following scientific inspection and received approval from the Medical Ethics Committee of Xiangya Hospital, Central South University.

### Establishment of SCI model and treatment

The mice were anesthetized by intraperitoneal injection of 0.3% pentobarbital sodium (75 mg/kg). Using the T10 spinous process as the center, the fur was removed, and a surgical area of about 3 cm × 1.5 cm was exposed. After disinfection, a 1 cm long incision was made in the middle of the dorsal skin of the mouse. The fascia tissue was cut layer by layer with ophthalmic scissors, and the lateral muscles of the spinous process were carefully separated to expose the vertebral body completely. Micro forceps were used to gently exfoliate the T10 spinous process and lamina, clearly exposing the spinal cord. Following the modified Allen’s method, a 10 g impactor rod struck vertically from a height of 2.5 cm. The observation of local hematoma in the spinal cord indicated the successful establishment of the SCI model. Subsequently, 4-0 sutures were used to stitch the muscles, fascia, and skin layer by layer, and the skin was disinfected with iodine. For the Sham group, only laminectomy was performed.

For adeno-associated virus (AAV) intervention, after exposing the spinal cord, 2 μL AAV-CNN1 or negative control AAV (NC-AAV) was locally injected into the T10 segment of the spinal cord using a 5 μL microinjection needle. The injection needle was kept in place for 2 min to promote full infiltration of AAV, then gently pulled out. After 2 weeks, when AAV began to be stably expressed, the SCI model was constructed by referring to the above method.

For dasatinib (D, MCE, United States) and quercetin (Q, MCE, United States) treatment, after establishing the SCI model, 5 μL hydrogel mixed with D + Q at concentrations of 60 μg/mL and 600 μg/mL, respectively, was placed on the surface of the injured spinal cord. Then, the hydrogel mixture was exposed to ultraviolet light for 5–9 s for solidification.

### bEnd.3 cell line culture and treatment

The bEnd.3 cell line was purchased from Procell Life Science&Technology Co.,Ltd., and was cultured with high glucose medium containing 10% fetal bovine serum (FBS) and 1% penicillin-streptomycin. H_2_O_2_ was used to induce cell senescence. We used culture medium to configure H_2_O_2_ at 0 μmol/L, 50 μmol/L, 100 μmol/L, 200 μmol/L, 400 μmol/L concentrations. Discard the culture medium and wash it with PBS before replacing it with the culture medium containing H_2_O_2_. After culturing for 12 h, detect cell senescence markers.

To establish a cell line stably expressing the target gene, lentivirus was constructed and screened with puromycin. UTX knockdown Lentivirus (UTX-KD) and corresponding negative control lentivirus (UTX-NC) were constructed by Shanghai Genechem Co., Ltd. CNN1 overexpression lentivirus (CNN1-OE) and control lentivirus (CNN1-NC) were constructed by OBiO Technology (Shanghai) Corp., Ltd. UTX overexpression lentivirus (UTX-UP) and control lentivirus (UTX-NC) were constructed by Suzhou Haixing Biological Technology Co., Ltd. After the cells reached an appropriate density, they were cultured with culture medium containing lentiviruses (at a MOI of 1:40) and auxiliary transfection reagents. The transfected cells were cultured for 24 h. When the cell growth concentration reached about 90%, stable cell lines were screened using puromycin for a continuous 7 days.

For D + Q treatment in vitro, 200 nmol/L dasatinib (D) and 20 μmol/L quercetin (Q) were added to the culture medium for 2 days, and then changed to normal culture medium until subsequent experiments started.

### Isolation and cultivation of BMECs

Mice were euthanized and subjected to disinfection by immersion in a 75% alcohol solution. The brain was extracted, and the brain stem, dura mater, and vascular tissue were removed. The cerebral cortex was then transferred to precooled DMEM medium and dissected using micro-scissors. Subsequently, centrifugation at 300 *g* for 5 min was performed, and the supernatant was discarded. A solution of 0.1% type II collagenase was introduced into the centrifuge tube. Following agitation and re-suspension, the mixture underwent digestion on a constant temperature shaker (37 °C) at 200 r/min for 1.5 h. The resulting suspension was subjected to centrifugation at 300 *g* for 5 min, and the supernatant was discarded. A 10 mL filtered solution of 20% BSA was added to the centrifuge tube, and the tissue was blown and resuspended. Subsequent centrifugation at 3 000 *g* for 20 min yielded the lower microvascular layer. The microvessels were digested with collagenase/dispersing enzyme for 30 min. BMECs were cultured in low-glucose DMEM containing 20% FBS, 0.5% FGF, 0.1% heparin sodium, and 1% penicillin-streptomycin.

### Immunofluorescent staining

To obtain spinal cord tissue, mice were anesthetized through an intraperitoneal injection of 0.3% sodium pentobarbital (75 mg/kg). Following the full exposure of the heart, a perfusion needle was inserted into the left ventricle, and 20 mL of physiological saline followed by 4% paraformaldehyde were sequentially infused at a rate of 4–5 mL/min. A 1.5 cm length of spinal segment was excised and dehydrated consecutively using 20% and 30% sucrose solutions. Subsequently, it was embedded in optimal cutting temperature (OCT) agent (Sakura, United States) for sectioning at a thickness of 16 μm.

After permeabilization and blocking, the sections were incubated with diluted primary antibodies at 4 °C overnight. On the following day, the primary antibodies were removed, and the sections were rinsed three times with a PBS solution. The corresponding diluted secondary antibodies were then added to the sections and incubated at room temperature for 1 h. Subsequently, the secondary antibodies were removed, and the sections were washed three times with PBS. 4,6-diaminyl-2-phenylindoles (DAPI, GeneTex, United States) were used to label cell nuclei. The images were captured using an APTOME Fluorescence Microscope (Zeiss, Germany). The antibodies used are listed in Table S1.

When cells reached a confluence of 85% in a 24-well plate, the culture medium was discarded, and the cells were washed with PBS three times. Subsequently, 500 μL of 4% paraformaldehyde was added to each well for fixation for 12 min. The paraformaldehyde was then washed away with PBS. Next, 200 μL of PBST containing 0.1% Triton-X100 was added to each well for permeabilization, and the cells were incubated for 20 min. Following permeabilization, the cells were blocked with a 3% BSA solution for 30 min. Subsequently, 200 μL of diluted primary antibodies were added to each well for overnight incubation at 4 °C. On the following day, the primary antibodies were removed, and the cells were washed with PBS. Diluted secondary antibodies were then added, and the cells were incubated at room temperature for 1 h. The cell nucleus was stained with DAPI. Images were captured using an inverted fluorescence microscope (Thermo Fisher, United States).

### SA-β-gal staining

The SA-β-gal kit (CST, United States) was thawed in advance. Initially, 200 μL of fixative was added to the specimen at room temperature for 15 min. Subsequently, the SA-β-gal working solution was prepared according to the manufacturer’s instructions, consisting of 10 μL of β-galactosidase solution A, 10 μL of β-galactosidase solution B, 930 μL of β-galactosidase solution C, and 50 μL of X-Gal solution. The pH value of the solution was adjusted to 6.8 using a pH meter. After washing with PBS twice, each specimen was treated with 200 μL of the working solution for incubation in a 37 °C oven overnight. On the following day, the working solution was removed, and the slices were photographed using an optical microscope (Leica, Germany).

For SA-β-gal staining of cells, when cell proliferation reached 75% confluence, the culture medium was removed, and 1 mL of 4% paraformaldehyde solution was added to each well to fix the cells for 15 min. The subsequent SA-β-gal staining process was identical to the procedure described above.

### Enzyme-linked immunosorbent assay (ELISA)

ELISA was employed to determine the concentration of senescence-associated secretory phenotype (SASP) factors (TNF-α, PAI-1, and CCL-5) in spinal cord tissue (Elabscience, China). Following euthanasia of the mouse, the spinal cord tissue containing the injured segment was obtained and rinsed with pre-cooled PBS solution. After homogenizing the tissue using a grinder, centrifugation at 4 °C, 5 000 *g* for 8 min was performed, and the supernatant was collected. The standard solution was prepared according to the instructions for constructing the standard curve. Subsequently, the sample was added to the ELISA plate and incubated at 37 °C for 90 min. Following this, biotinylated antibody diluent was added, and the incubation continued at 37 °C for 1 h. After terminating the reaction, a microplate reader (Thermo Fisher, United States) was utilized to measure absorbance at a wavelength of 450 nm.

### Real-time fluorescence quantitative polymerase chain reaction (qRT-PCR)

Total RNA of spinal cord tissue or bEnd.3 cells were extracted using Trizol agent (Sigma, United States). Add chloroform in the ratio of Trizol: chloroform (5:1). Leave the mixture on ice for 10 min after shaking. Then centrifuge at 4 °C at 12 000 r/min for 15 min. Carefully absorb the top supernatant and transfer it to a new 1.5 mL EP tube. Add an equal amount of isopropanol to the EP tube, turn it upside down 10 times. Then centrifuge at 12 000 r/min for 15 min. Discard the supernatant and wash the RNA with 75% alcohol. Then 20 μL enzyme-free water was added and gently blown to fully dissolve the RNA. After detecting the RNA concentration using a Nanodrop (Thermo Fisher, United States), a Reverse Transcription System (Promega, United States) was used to reverse transcription of RNA into cDNA. GoTaq® qPCR Master Mix (Promega, United States) was used for real-time quantitative PCR. PCR results were analyzed by 2^−ΔΔCt^ method. GAPDH was used as internal reference. Primers used were listed in Table S2.

### Western blot (WB)

Total proteins from spinal cord tissue or bEnd.3 cells were extracted using RIPA lysis (Sigma, United States) and protease inhibitor (Sigma, United States). The tissue or cells were fully lysed after ultrasonic treatment for 120 s. Subsequently, the lysate was centrifuged at 4 °C and 12 000  r/min for 15 min, and the supernatant was collected. The protein concentration was determined using the BCA protein detection kit and a microplate reader (Thermo Fisher, United States). A 10% gel was utilized to separate proteins with different molecular weights. After adding samples and markers, the voltage was adjusted to 90 V for electrophoresis until the protein bands were completely separated. The proteins were then electro-transferred to a polyvinylidene fluoride (PVDF) membrane at 300 mA for 90 min. The PVDF membrane was blocked with 5% non-fat milk for 1.5 h and incubated with corresponding primary antibodies at 4 °C overnight. On the following day, the primary antibodies were removed, and the membrane was rinsed with TBST solution three times. Subsequently, secondary antibodies were added for incubation at room temperature for 1 h. Bands were covered with ECL chemiluminescence (Millipore, United States) and visualized using a chemiluminescence instrument (Bio-Rad, United States). Actin was used as an internal reference. Antibodies used are listed in Table S1.

### Tube formation and transwell migration

bEnd.3 cells were subjected to serum starvation by replacing the culture medium with FBS-free medium. Following trypsin digestion and centrifugation, bEnd.3 cells were adjusted to a concentration of 80 000 cells/mL with FBS-free culture medium. Matrigel (Corning, United States) was evenly applied to cover a 96-well plate, and the plate was then incubated in a constant temperature incubator for 30 min. Subsequently, 100 μL of the cell suspension was added to each well of the 96-well plate and cultured in a constant temperature incubator for 6 h. The canalization of cells was observed using an optical microscope (Leica, Germany) and quantified using Image J software.

For the assessment of the vertical migration capacity of bEnd.3 cells, Transwell plates (Corning, United States) were employed. The lower chamber was filled with 600 μL of culture medium containing 10% FBS, and 100 μL of the cell suspension was added to the upper chamber. After incubating in a constant temperature incubator for 12 h, the transwell chambers were removed, fixed with 4% paraformaldehyde for 15 min, and washed three times. Following staining with 1% crystal violet solution for 10 min, the number of cells that migrated from the upper chamber to the lower chamber was observed using a microscope (Leica, Germany), and Image J software was used for counting.

### Scratch wound healing assay

bEnd.3 cells were seeded in six-well plates, with approximately 200 000 cells in each well. Once the cells completely covered the surface of the six-well plate, they were subjected to a 6 h starvation period. Using a 200 μL tip, lines perpendicular to the cell surface were drawn, and the detached cells were washed off with PBS. Subsequently, the cells were replenished with FBS-free culture medium and cultured for an additional 12 h in a constant temperature incubator. Cell migration was observed using optical microscopy at 0 and 12 h after creating the scratches, and Image J software was employed to calculate the migration distance.

### PCR-array

PCR-array kits were customized by Wcgene Biotech, Shanghai, China. bEnd.3 cells were used to detect SASP expression after H_2_O_2_ treatment. BMECs were used to screen differentially expressed epigenetic factors after H_2_O_2_ treatment. cDNA mix solution was prepared according to the manufacturer’s instructions. The PCR-array plate was rapidly centrifuged for 20 s, and 9 μL cDNA mix solution was added to each well. The PCR process was set according to the following procedures: activation of polymerase at 95 °C for 2 min, denaturation at 95 °C for 15 s and annealing extension at 60 °C for 1 min (40 cycles).

### Basso mouse scale (BMS) scores

BMS scores were performed to explore motor function of mice, as previously described.^55^ Prior to the test, mice were placed on the experimental table to acclimate to the environment. Two well-trained observers simultaneously observed hind limb movements and trunk stability for each mouse over a 4-minute period. The final score at each time point was determined by calculating the average score from the two observers (0 indicated complete paralysis, and 9 indicated normal motor function). BMS scores were assessed before the injury, and at 1 day, 3 days, 7 days, 2 weeks, 3 weeks, and 4 weeks post-injury.

### Neuroelectrophysiological evaluation

The Neuroelectrophysiological evaluation was used to detect the neural conductivity in mice, and performed as previously described.^56^ Briefly, mice were anesthetized via intraperitoneal injection of 0.3% sodium pentobarbital (75 mg/kg). A stereotaxic device was utilized to determine the skull location, with bregma set as the origin. Two stimulation electrodes were punctured into the skin and positioned on the skull surface (1 mm on the caudal side and 0.5 mm on the lateral side, −4 mm on the caudal side and 0.6 mm on the lateral side). Recording electrodes were inserted into the tibialis anterior muscle of the contralateral posterior limb, and a reference electrode was placed in the subcutaneous tissue between the stimulation and recording electrodes. Electrical stimulation was applied at a voltage of 3 V and a frequency of 333 Hz, repeated every 5 s. The evoked potential was determined by the peak and trough voltage, while the latent period was defined as the time from the initial stimulation to the occurrence of a dramatic change in the waveform.

### Evaluation of neurogenic bladder

Bladder function was assessed by measuring the bladder diameter and detrusor muscle thickness through HE staining. Following anesthesia, mice were perfused with physiological saline and 4% paraformaldehyde. The bladder was photographed, and the diameter at its widest part was measured. For detrusor muscle thickness measurement, the bladder underwent gradient dehydration with varying concentrations of anhydrous ethanol. Subsequently, the bladder tissue was embedded in paraffin and sectioned into 8 μm slices. Paraffin sections were dewaxed, hydrated, and stained with hematoxylin for 5 min. Excess hematoxylin solution was washed off, followed by staining with eosin solution for 1 min. After removing excess eosin solution, the slices were dehydrated and sealed with neutral resin.

### RNA-seq

RNA-seq analysis was conducted by Seqhealth Technology (Wuhan, China) with four biological replicate samples per group. Primary brain microvascular endothelial cells (BMECs) were isolated from 8-week-old UTX^−/−^ and UTX^flox/flox^ female mice by flow cytometry. Following treatment with 200 μmol/L H_2_O_2_ for 12 h to induce senescence, BMECs underwent RNA extraction. Oligo (dT) was then employed to enrich mRNA, and the enriched mRNA underwent fragmentation, reverse transcription, and PCR amplification to generate a sequencing library. Subsequently, Illumina Hiseq was utilized for RNA sequencing. After preprocessing, quality control, and sequence alignment of the data, protein-coding genes annotated by the genome were obtained. Differentially expressed genes were identified using the criteria of an absolute logFC > 1 and *P* < 0.05. These genes were selected for subsequent Gene Ontology (GO) and Kyoto Encyclopedia of Genes and Genomes (KEGG) analyses. The top 10 down-regulated genes were listed in Table S3.

### ChIP-qPCR

To investigate the targeted binding relationship between UTX and H3K27 of the CNN1 promoter, ChIP-qPCR detection was conducted. bEnd.3 cells with stable UTX overexpression were obtained through Lentivirus transfection. Formaldehyde was added to the cell culture medium, followed by incubation at room temperature for 10 min, and glycine was added to terminate crosslinking. After discarding the culture medium and washing with precooled PBS, digestive fluid was added to the cells for ultrasonic lysis. Ten microliters of supernatant were taken as input, and the remaining supernatant was used for IP. The IP group received either H3K27me3 antibody or IgG antibody, while no antibody was added to the input group. After eluting DNA from the antibodies, a DNA purification kit (Magen, China) was used to recover the eluted DNA. The primers for ChIP-qPCR are listed in Table S4.

### Statistical analysis

Statistical analyses were performed using GraphPad Prism 8 software. A student’s *t* test was utilized for comparisons between two groups, while differences among multiple groups were assessed using one-way ANOVA. Tukey post-test was employed for pairwise comparisons in the case of multiple groups. BMS scores at different time points within each group were analyzed using repeated-measure two-way ANOVA. Results were presented as mean ± standard deviation (Mean ± SD), and when *P* < 0.05, it is considered statistically significant.

### Supplementary information

Supplementary Information

## Data Availability

All data supporting the conclusions of this manuscript are provided in the text and figures. Please contact the author for data requests.
