# Supplementary material for: Kdm6a-CNN1 axis orchestrates epigenetic control of trauma-induced spinal cord microvascular endothelial cell senescence to balance neuroinflammation for improved neurological repair
Source: Bone Res. 2024 Mar 25;12:19. doi: 10.1038/s41413-024-00323-x (PMC10963366; doi:10.1038/s41413-024-00323-x)
Supplement: Supplementary file 1 — Supplementary Information [file 41413_2024_323_MOESM1_ESM.docx]

**Supplemental Figures**


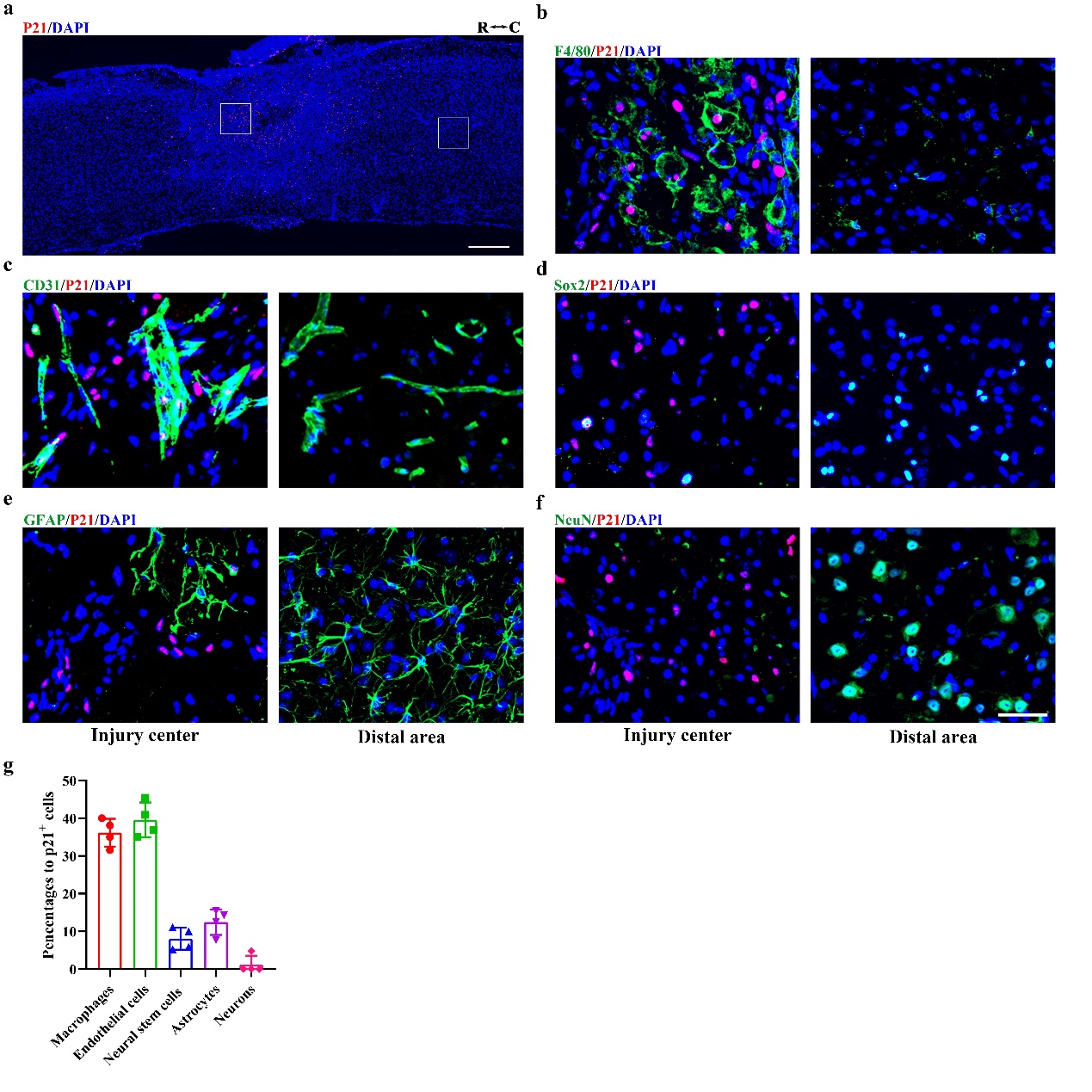


**Figure S1 Senescence phenotype of different cell types after SCI.** (a) Representative images of p21 (red) staining 14 days after SCI. Scale bar: 200μm. The white squares represent the selected area of interest in the center and periphery of the injury. R represents rostral, C represents caudal. (b) Representative images of p21 (red) staining and F4/80 (green) in injury center and distal area. (c) Representative images of p21 (red) staining and CD31 (green) in injury center and distal area. (d) Representative images of p21 (red) staining and Sox2 (green) in injury center and distal area. (e) Representative images of p21 (red) staining and GFAP (green) in injury center and distal area. (f) Representative images of p21 (red) staining and NeuN (green) in injury center and distal area. Scale bar: 20μm. (g) Quantification of percentages of senescent cells to p21 positive cells in (b-f). n=4.


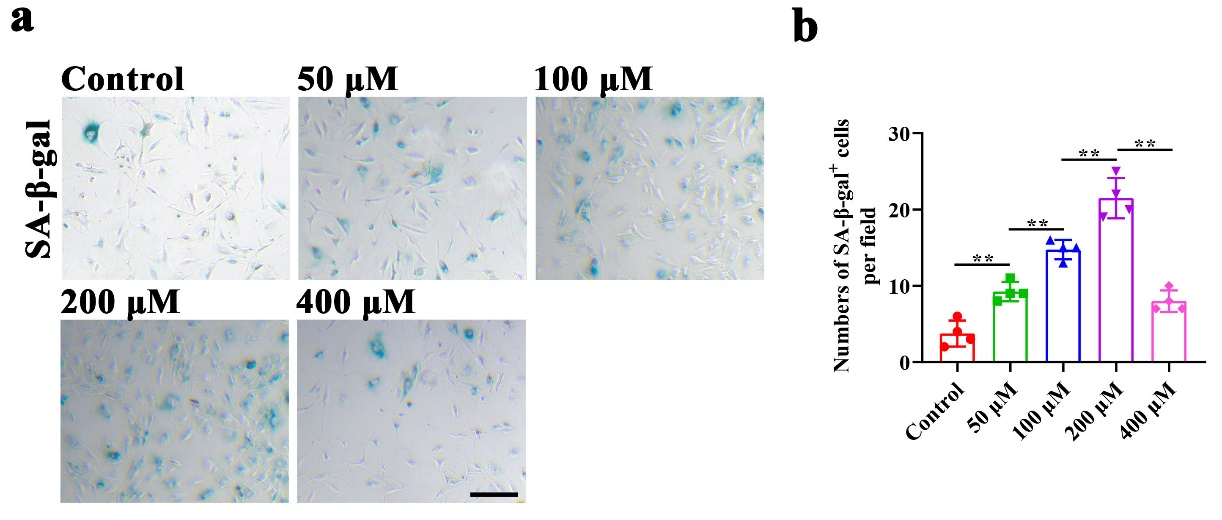


**Figure S2 200μM H_2_O_2_ was the optimal concentration to induce cellular senescence.** (a) SA-β-gal staining of bEnd.3 cells under treatment with different concentrations of H_2_O_2_. Scale bar: 100μm. (b) Quantification of SA-β-gal^+^ cells in (a). n=4. Data are presented as the mean ± SD. **P < 0.01.


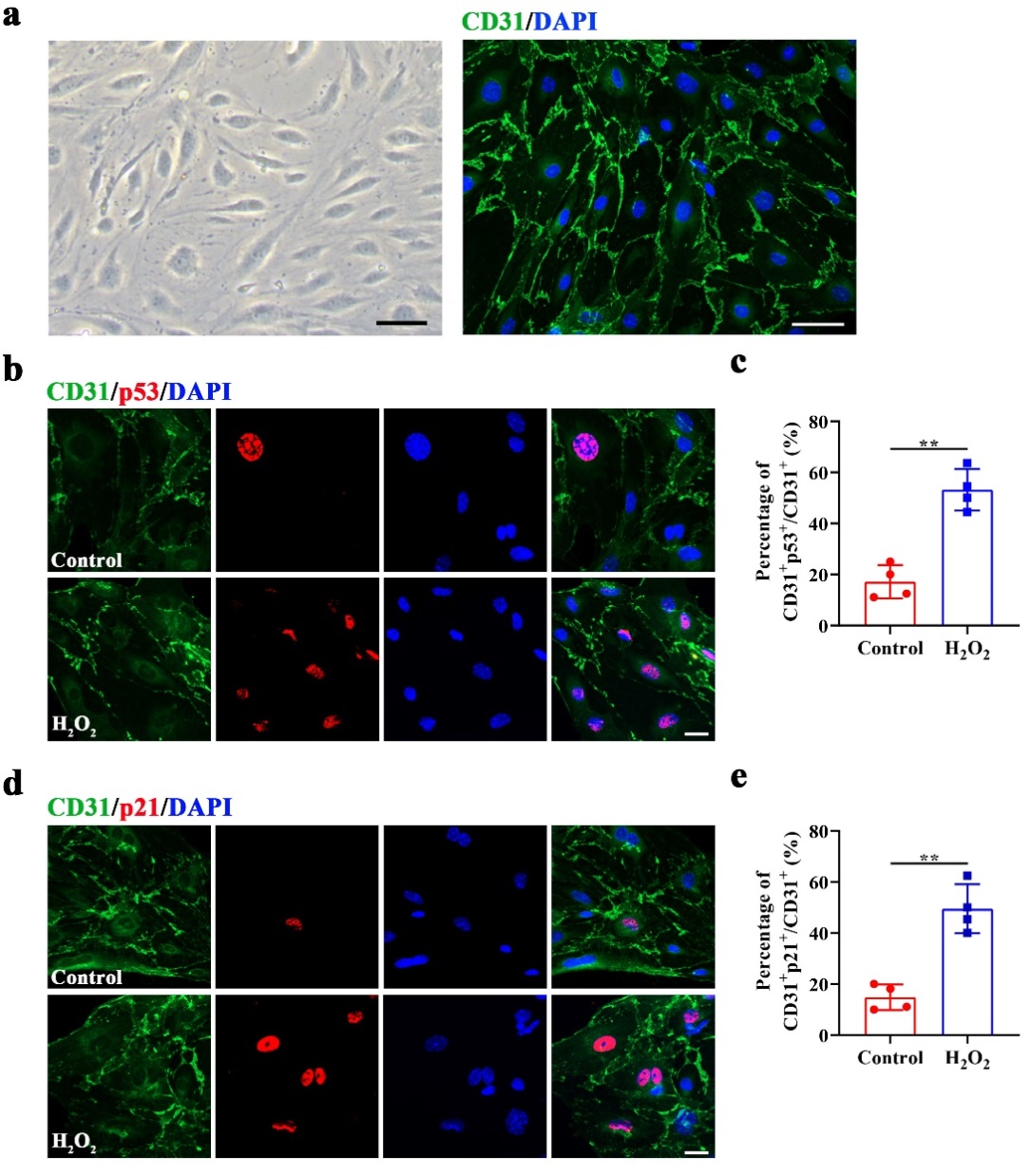


**Figure S3 H_2_O_2_ induced BMECs senescence.** (a) Optical microscope image and representative fluorescent image of BMECs. Scale bar: 50μm. (b) Representative images of CD31 (green) and p53 (red) in control and H_2_O_2_ treated BMECs. Scale bar: 20μm. (c) Quantification of percentages of CD31^+^p53^+^/CD31^+^ cells in (b). n=4. (d) Representative images of CD31 (green) and p21 (red) in control and H_2_O_2_ treated BMECs. Scale bar: 20μm. (e) Quantification of percentages of CD31^+^p21^+^/CD31^+^ cells in (d). n=4. Data are presented as the mean ± SD. **P < 0.01.

**
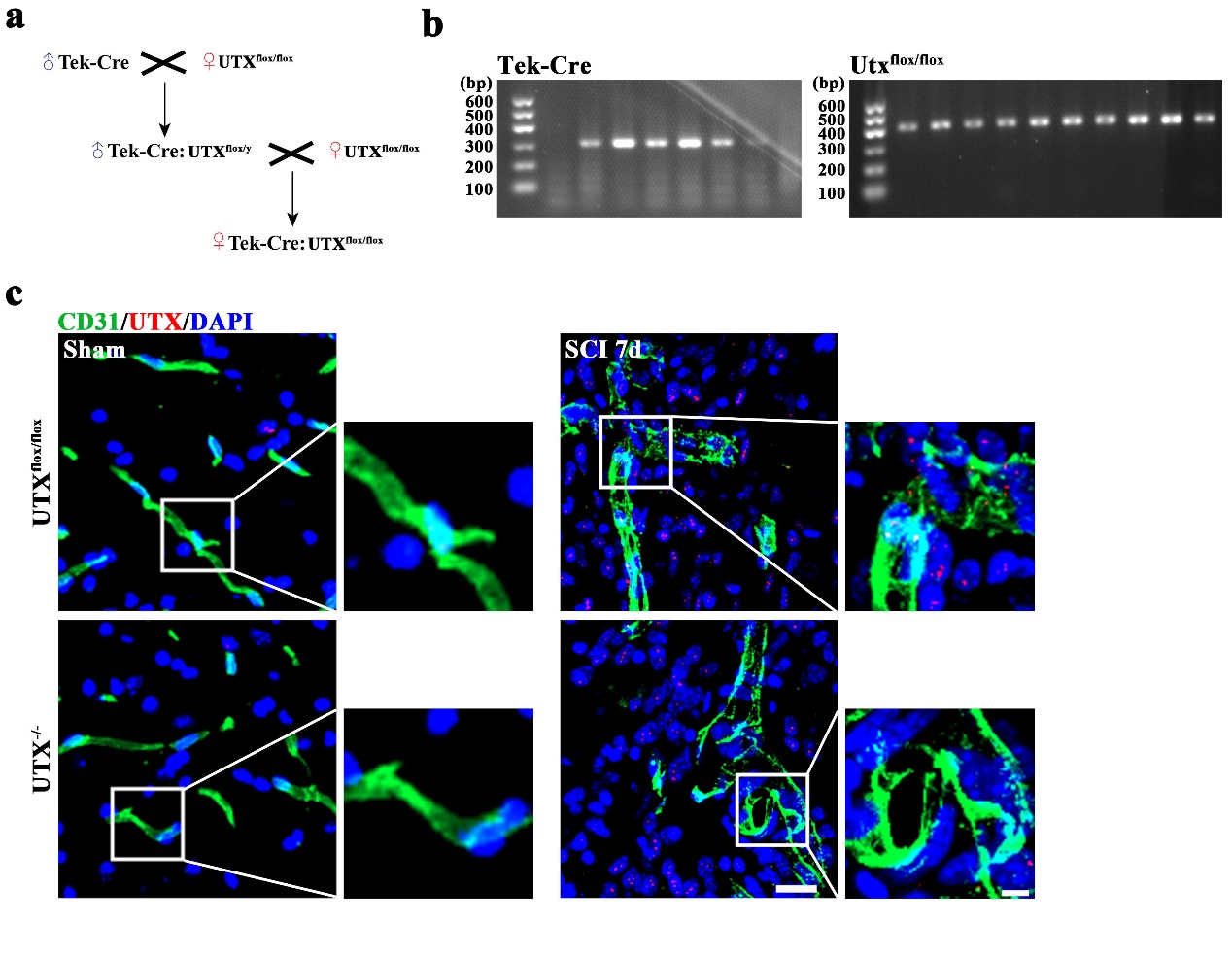
**

**Figure S4 Construction and identification of vascular endothelial cell UTX conditional knockout mice.** (a) Schematic of breeding of vascular endothelial cell UTX-conditional knockout mice (Tek-Cre: UTX^flox/flox^). (b) DNA identification of Tek-Cre and UTX^flox/flox^. (c) Representative images of CD31 (Green) and UTX (Red) staining before injury and at 7days post SCI in UTX^flox/flox^ and UTX^-/-^ mice. Scale bar: 20μm. Scale bar for magnified images: 5μm.


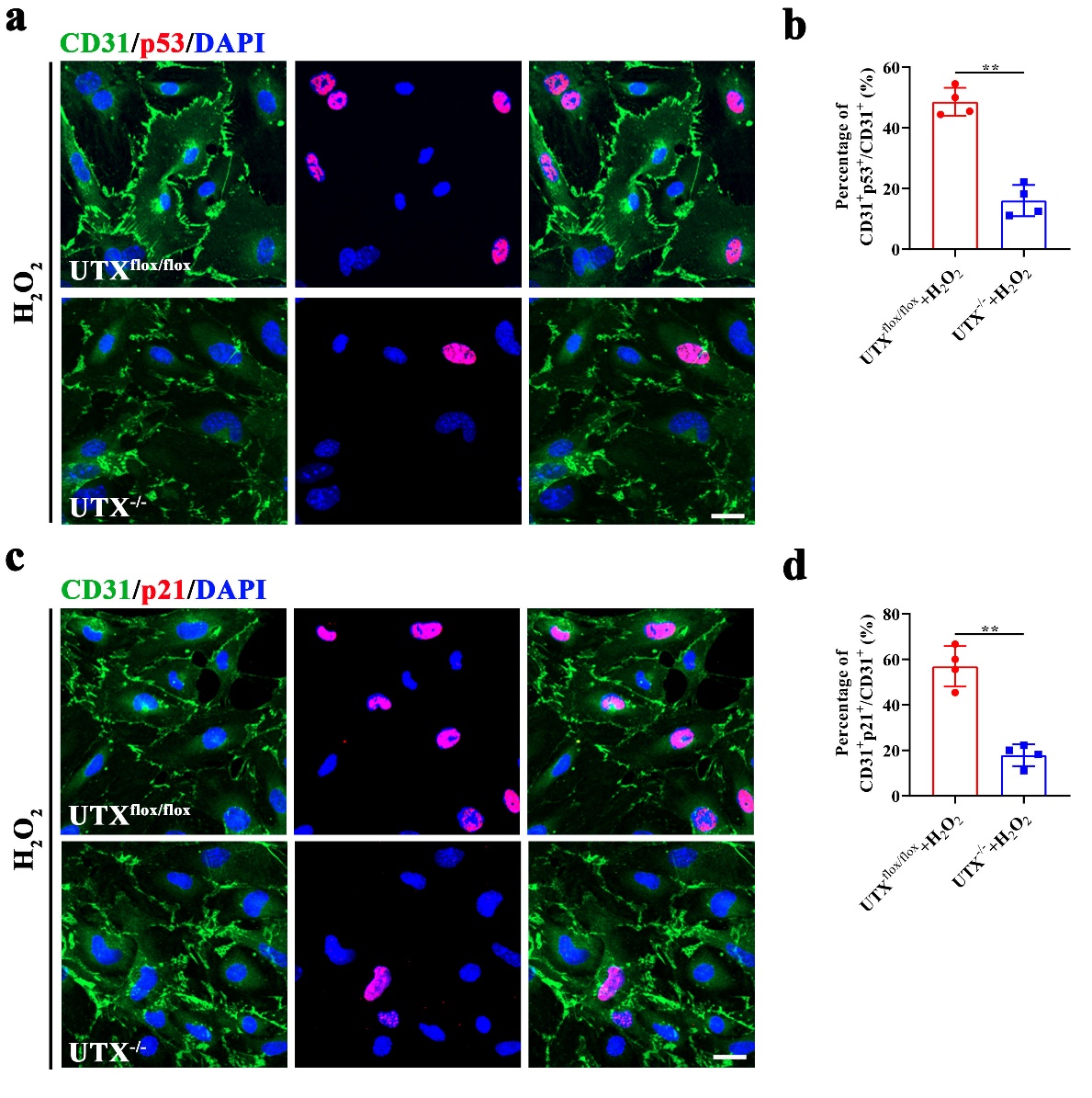


**Figure S5 UTX knockout depressed BMECs senescence in vitro.** (a) Representative images of CD31 (green) and p53 (red) in UTX^flox/flox^ and UTX^-/-^ BMECs after H_2_O_2_ treatment. Scale bar: 20μm. (b) Quantification of percentages of CD31^+^p53^+^/CD31^+^ cells in (a). n=4. (c) Representative images of CD31 (green) and p21 (red) in UTX^flox/flox^ and UTX^-/-^ BMECs after H_2_O_2_ treatment. Scale bar: 20μm. (d) Quantification of percentages of CD31^+^p21^+^/CD31^+^ cells in (c). n=4. Data are presented as the mean ± SD. **P < 0.01.


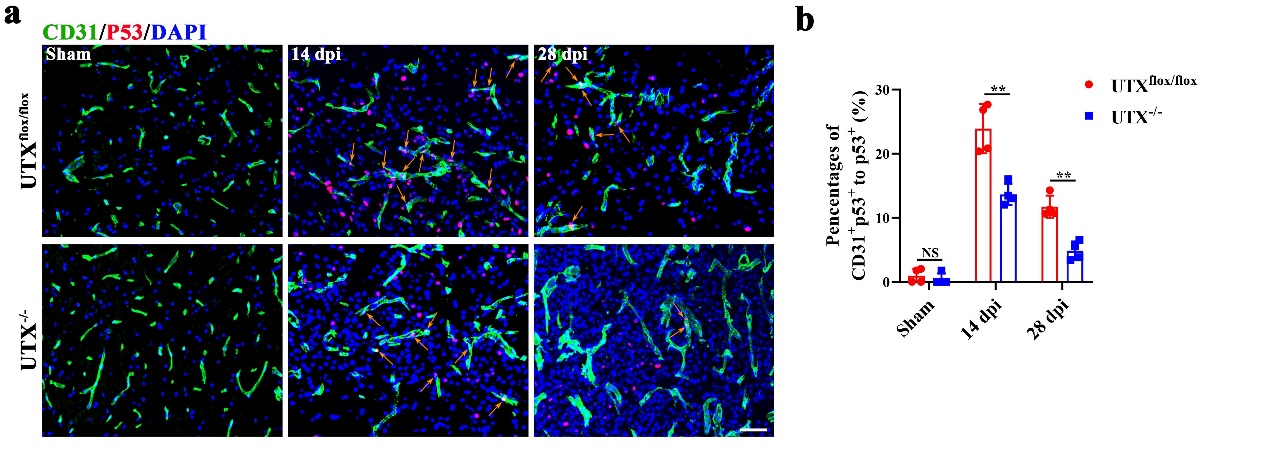


**Figure S6 UTX deletion decreased SCMECs senescence after SCI.** (a) Representative images of CD31 (green) and p53 (red) staining before injury and at 14days, 28days post SCI in UTX^flox/flox^ and UTX^-/-^ mice. Scale bar: 50μm. The orange arrow indicates CD31^+^p53^+^ cell. (b) Quantification of percentages of CD31^+^p53^+^/CD31^+^ cells in (a). n=4. Data are presented as the mean ± SD. **P < 0.01. ns=not significant.


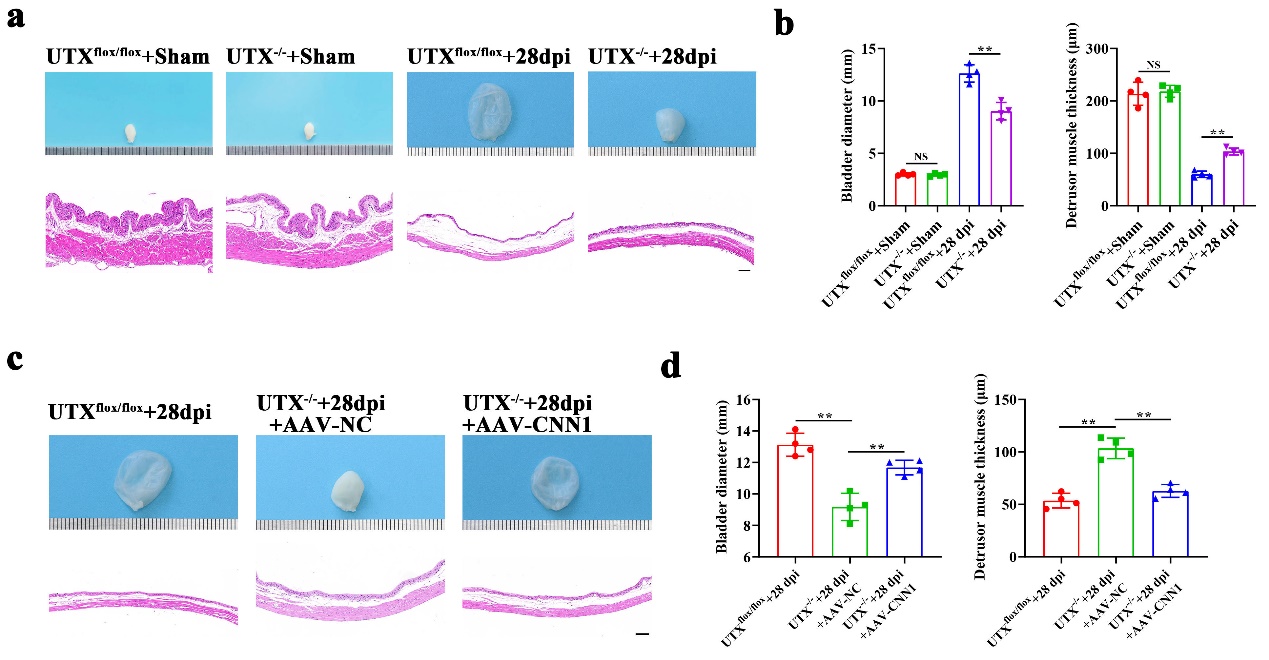


**Figure S7 Bladder contractility.** (a) Gross images and HE staining of bladder in UTX^flox/flox^ and UTX^-/-^ mice before injury and 28 days after SCI. Scale bar for lower panel: 100μm. (b) Quantification of bladder diameter and detrusor muscle thickness in (a). n=5. (c) Gross images and HE staining of bladder in UTX^flox/flox^, UTX^-/-^ + AAV-NC and UTX^-/-^ + AAV-CNN1 mice. Scale bar for lower panel: 100μm. (d) Quantification of bladder diameter and detrusor muscle thickness in (c). n=5. Data are presented as the mean ± SD. **P < 0.01. ns=not significant.


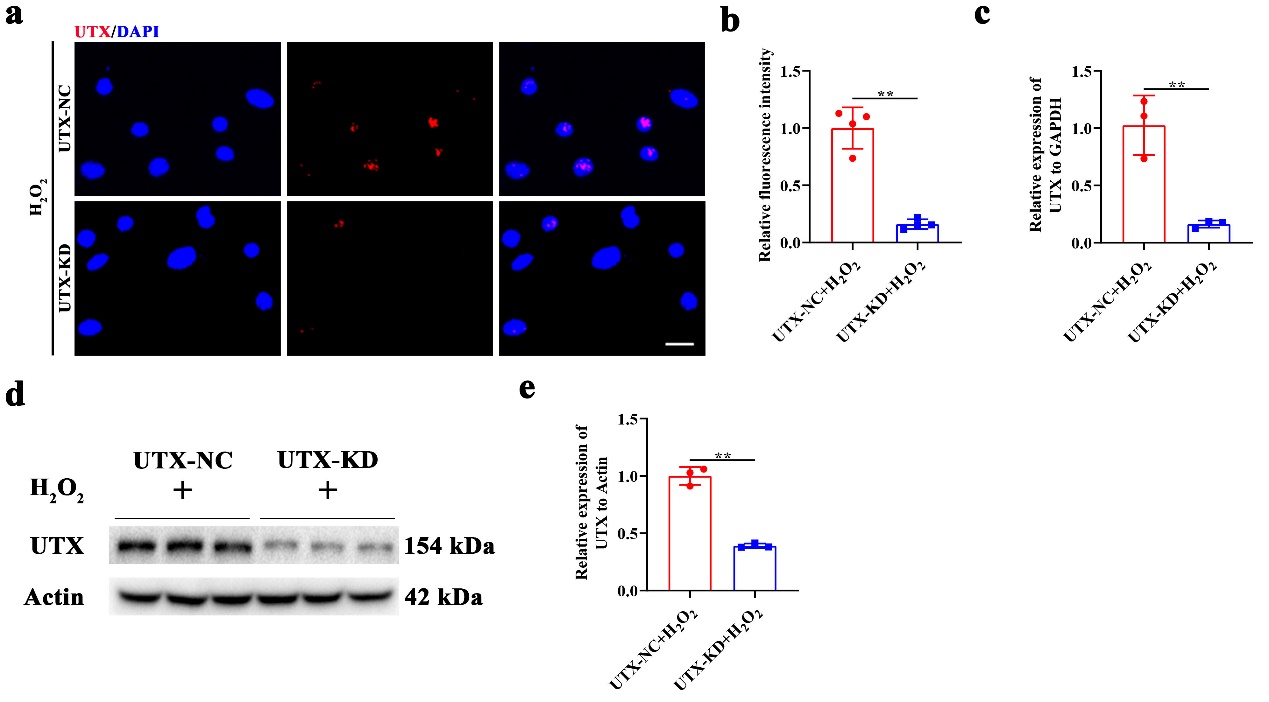


**Figure S8 Efficiency of UTX knockdown in ECs in vitro.** (a) Representative images of UTX staining in UTX-NC and UTX-KD groups after H_2_O_2_ treatment. Scale bar: 20μm. (b) Quantification of relative UTX intensity in (a). n=4. (c) Relative expression of UTX in UTX-NC and UTX-KD treated bEnd.3 cells under H_2_O_2_ intervention. n=3. (d) Western blot of UTX expression in UTX-NC and UTX-KD treated bEnd.3 cells under H_2_O_2_ intervention. (e) Quantification of UTX expression in (d). n=3. Data are presented as the mean ± SD. **P < 0.01.


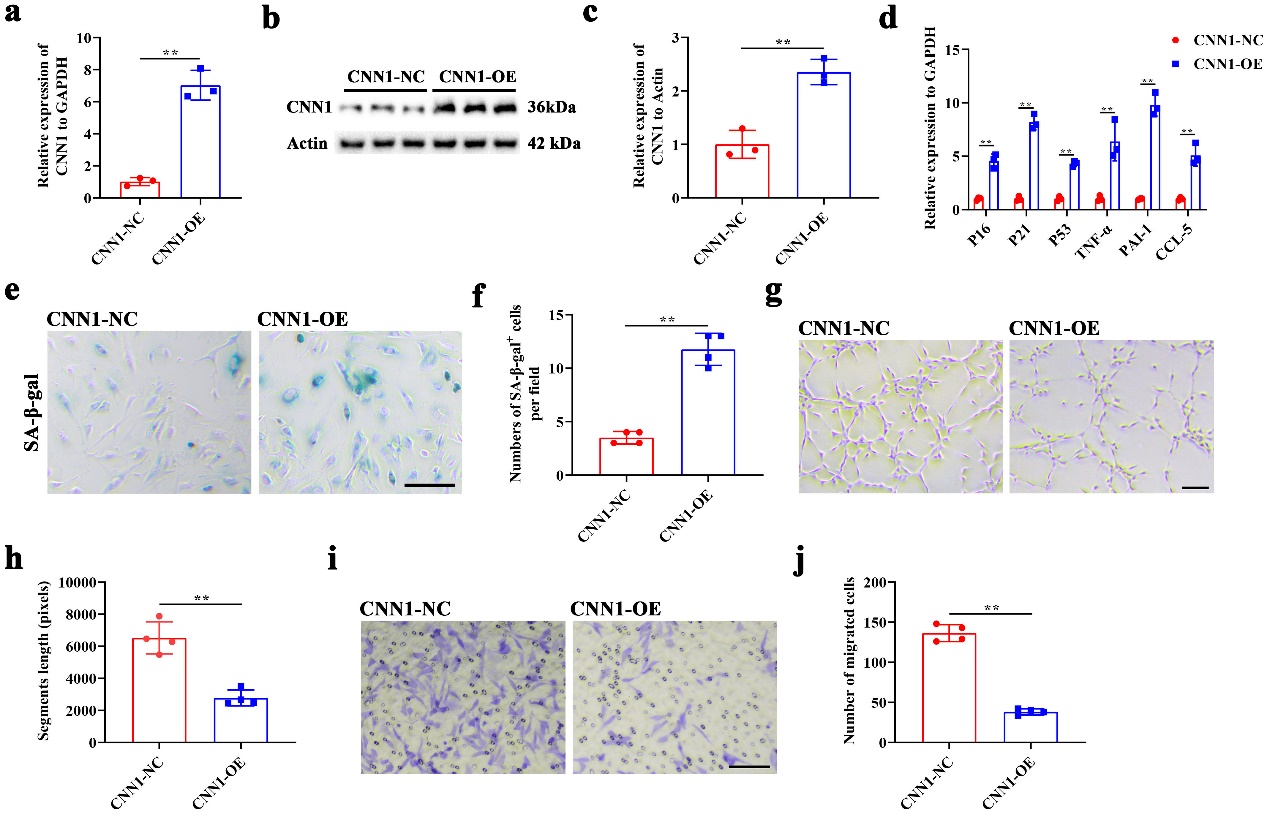


**Figure S9 CNN1 promoted endothelial cell senescence and impaired cellular biological function.** (a) Relative expression and quantification of CNN1 in CNN1-NC and CNN1-OE treated bEnd.3 cells by qRT-PCR. n=3. (b) Western blot of CNN1 expression in CNN1-NC and CNN1-OE treated bEnd.3 cells. (c) Quantification of CNN1 expression in (b). n=3. (d) Relative expression of P16, P21, P53, TNF-α, PAI-1 and CCL-5 in CNN1-NC and CNN1-OE treated bEnd.3 cells by qRT-PCR. n=3. (e) Representative images of SA-β-gal staining in CNN1-NC and CNN1-OE treated bEnd.3 cells. Scale bar: 100μm. (f) Quantification of SA-β-gal+ cells in (e). n=4. (g) Representative images of bEnd.3 canaliculization in CNN1-NC and CNN1-OE treated groups. Scale bar: 100μm. (h) Quantification of segments lengths in (g). n=4. (i) Representative images of transwell migration in CNN1-NC and CNN1-OE treated bEnd.3 cells. Scale bar: 100μm. (j) Quantification of migrated cells in (i). n=4. Data are presented as the mean ± SD. **P < 0.01.


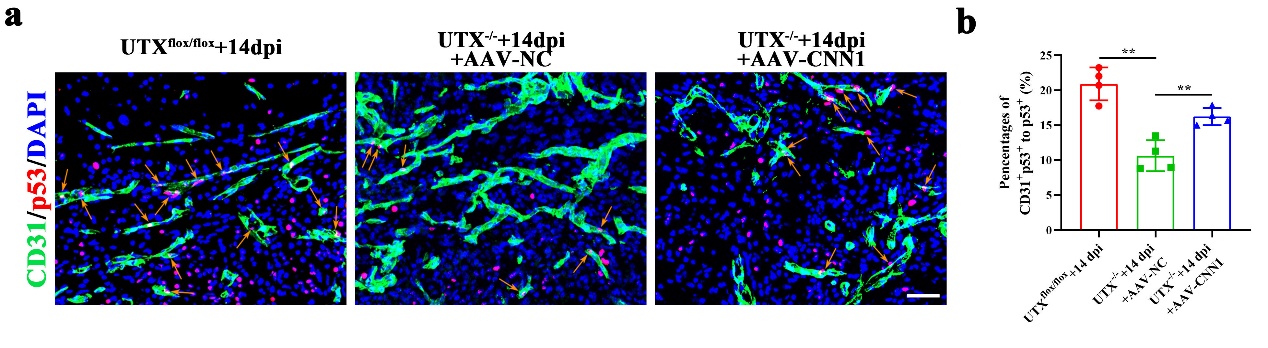


**Figure S10 UTX deletion in endothelial cells decreased senescence in SCMECs by downregulating CNN1.** (a) Representative images of CD31 (green) and p53 (red) staining 14 days post SCI in UTX^flox/flox^, UTX^-/-^ + AAV-NC and UTX^-/-^ + AAV-CNN1 groups. Scale bar: 50μm. The orange arrow indicates CD31^+^p53^+^ cell. (b) Quantification of percentages of CD31^+^p53^+^/CD31^+^ cells in (a). n=4. Data are presented as the mean ± SD. **P < 0.01.


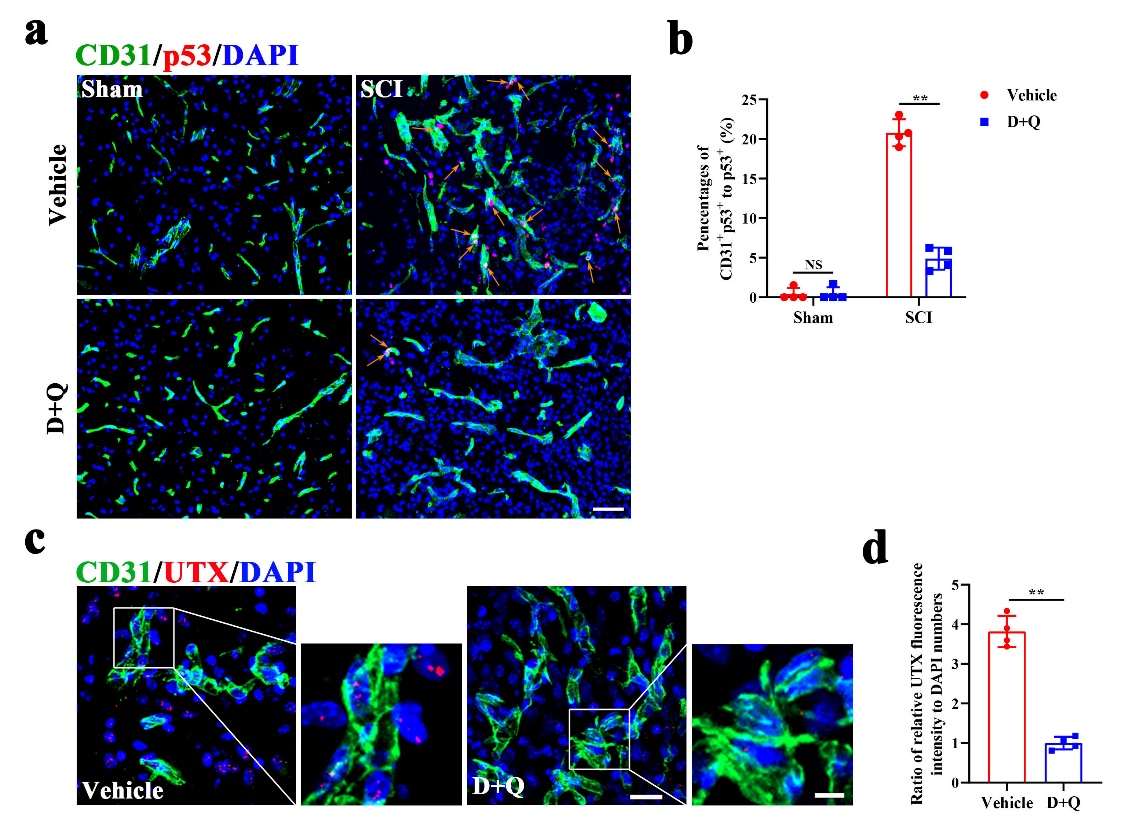


**Figure S11 Senolytic drugs downregulated UTX expression and decreased SCMECs senescence.** (a) Representative images of CD31 (green) and p53 (red) staining before and after SCI in vehicle and D+Q treated mice. Scale bar: 50μm. The orange arrow indicates CD31^+^p53^+^ cell. (b) Quantification of percentages of CD31^+^p53^+^/CD31^+^ cells in (a). n=4. (c) Representative fluorescent images of CD31 (green) and UTX (red) after SCI in vehicle and D+Q treated mice. Scale bar: 20μm. Scale bar for magnified images: 5μm. (d) Quantification of (c). Data are presented as the mean ± SD. **P < 0.01. ns=not significant.

Table S1 Antibodies information

| Antibodies | Application and Dilution | Catalog numbers and species |
| --- | --- | --- |
| CD31 | IF, 1:200 | R＆D (FAB3628G-100, Goat) |
| UTX | IF, 1:400 | Millipore (SAB2102665, Rabbit) |
| UTX | WB, 1:1000 | CST (33510S, Rabbit) |
| Actin | WB, 1:20000 | Proteintech (66009-1-Ig, Mouse) |
| p53 | WB,1:750 | Wanleibio (WL01919, Rabbit) |
| p53 | IF, 1:400 | Proteintech (60283-2-Ig, Mouse) |
| p21 | IF, 1:200; WB, 1:2000 | Abcam (ab109520, Rabbit) |
| p16 | WB, 1: 500 | Zenbio (R22878, Rabbit) |
| CNN1 | IF, 1:200; WB, 1:1000 | Proteintech (13938-1-AP, Rabbit) |
| LaminB1 | WB, 1:20000 | Proteintech (66095-1-Ig, Mouse) |
| H3K27me3 | WB, 1:1000 | CST (9733T, Rabbit) |
| Histone 3 | WB, 1:2000 | Transgen (HL102, Mouse) |

IF: immunofluorescence; WB: western blot

Table S2 Primers for qRT-PCR

| Genes | Primers (5’-3’) |
| --- | --- |
| P16 | F：CGCAGGTTCTTGGTCACTGT |
|  | R：TGTTCACGAAAGCCAGAGCG |
| P21 | F：CCTGGTGATGTCCGACCTG |
|  | R：CCATGAGCGCATCGCAATC |
| P53 | F：GTCACAGCACATGACGGAGG |
|  | R：TCTTCCAGATGCTCGGGATAC |
| TNF-α | F：GACGTGGAACTGGCAGAAGAG |
|  | R：TTGGTGGTTTGTGAGTGTGAG |
| PAI-1 | F：TTCAGCCCTTGCTTGCCTC |
|  | R：ACACTTTTACTCCGAAGTCGGT |
| CCL-5 | F：GCTGCTTTGCCTACCTCTCC |
|  | R：TCGAGTGACAAACACGACTGC |
| UTX | F：AAGGCTGTTCGCTGCTACG |
|  | R：GGATCGACATAAAGCACCTCC |
| CNN1 | F：TCTGCACATTTTAACCGAGGTC |
|  | R：GCCAGCTTGTTCTTTACTTCAGC |
| Wnt7a | F: CCTTGTTGCGCTTGTTCTCC |
|  | R: GGCGGGGCAATCCACATAG |
| Acta2 | F: CCCAACTGGGACCACATGG |
|  | R: TACATGCGGGGGACATTGAAG |
| Acta1 | F: CCCAAAGCTAACCGGGAGAAG |
|  | R: CCAGAATCCAACACGATGCC |
| Krt80 | F: GTCTCCGAAGAACTGAACTGG |
|  | R: TCACTTGGGCTGTAAGGTCTTT |
| Lmod1 | F: AGGTGAGTGAAGACCCCGA |
|  | R: TGTCCGTTTGATTCCTCTGCC |
| Fgd3 | F: GGATCGAGTCCAGAAAGTCGT |
|  | R: CAGCTTGCAGCGATACCTCC |
| Psca | F: GGACCAGCACAGTTGCTTTAC |
|  | R: GTAGTTCTCCGAGTCATCCTCA |
| GAPDH | F：GGTTGTCTCCTGCGACTTCA |
|  | R：TGGTCCAGGGTTTCTTACTCC |

Table S3 The top 10 down-regulated genes by RNA-seq

| Gene ID | LogFC | p-value |
| --- | --- | --- |
| Wnt7a | -9.11896 | 1.53E-10 |
| Acta2 | -8.308997 | 4.03E-11 |
| CNN1 | -6.697288 | 2.93E-08 |
| Acta1 | -5.91621 | 1.75E-09 |
| Krt80 | -5.768205 | 6.85E-08 |
| Lmod1 | -5.64302 | 7.01E-08 |
| Fgd3 | -5.601243 | 7.21E-12 |
| Psca | -5.53348 | 9.78E-08 |
| Lce1g | -5.410003 | 5.69E-07 |
| Myl9 | -5.163415 | 2.04E-06 |

Table S4 The primers for ChIP-qPCR

| Sites | Primers (5’-3’) |
| --- | --- |
| CNN1-promoter 1 | F：GCCCTCAGGAAGAGATCCAC |
|  | R：AGCGCCTCCGTACTGGTATC |
| CNN1-promoter 2 | F：GAAATGAGGTAGGCGGCAAG |
|  | R：CGAGTCCCGGAAGAAGTACG |
| CNN1-promoter 3 | F：CCACCTCCGAGTGCTAACCT |
|  | R：ACCTAACCACAGGCCCACAG |
